# Supplementary material for: Deep coverage and extended sequence reads obtained with a single archaeal protease expedite de novo protein sequencing by mass spectrometry
Source: Cell Syst. 2026 Apr 15;17(4):None. doi: 10.1016/j.cels.2026.101536 (PMC13083267; doi:10.1016/j.cels.2026.101536)
Supplement: Document S1. Figures S1–S22 and Tables S1 and S2 [file mmc1.pdf]

Cell Systems, Volume *17*

Supplemental information

Deep coverage and extended sequence reads obtained with a single archaeal protease expedite *de novo* protein sequencing by mass spectrometry

Laura Pérez Pañeda, Tereza Kadavá, Tatiana M. Shamorkina, Douwe Schulte, Patrick Pribil, Sibylle Heidelberger, Allison Michele Narlock-Brand, Steven M. Yannone, Joost Snijder, and Albert J.R. Heck

Supporting Information

Deep Coverage and Extended Sequence Reads Obtained with a Single Archaeal Protease Expedite *de novo* Protein Sequencing by Mass Spectrometry

Laura Pérez Pañeda[*, 1], Tereza Kadavá[*, 1], Tatiana M. Shamorkina[*, 1], Douwe Schulte[1], Patrick Pribil[2], Sibylle Heidelberger[3], Allison Michele Narlock-Brand[4], Steven M. Yannone[4], Joost Snijder[1], and Albert J. R. Heck[1, 5, +]

*These authors contributed equally

[1]Biomolecular Mass Spectrometry and Proteomics, Bijvoet Center for Biomolecular Research and Utrecht Institute for Pharmaceutical Sciences, University of Utrecht, Padualaan 8, Utrecht 3584 CH, the Netherlands and Netherlands Proteomics Center, Padualaan 8, Utrecht 3584 CH, the Netherlands

[2]SCIEX, 71 Four Valley Drive, Concord, ON, Canada L4K 4V8

[3]SCIEX, UK Alderley Park, 21 Mereside, Macclesfield UK SK10 4TG

[4]Cinder Biological, Inc., San Leandro, California 94577, United States

[5]Lead contact

+Correspondence: a.j.r.heck@uu.nl

Contents

Figures

S1. Optimization of kinetic energy (KE) parameters for the EAciD method
S2. Representative fragmentation spectra generated with CID, EAD and EAciD, normalized
S3. Representative fragmentation spectra generated with CID, EAD and EAciD, magnified
S4. EAciD performs especially well on long-read peptides
S5. EAciD provides high peptide coverage across all peptide charges
S6. Confirmation that EAciD can be used to distinguish Leu from Ile
S7. Sequence logos for (A) Krakatoa and (B) Vesuvius protease cleavage specificities
S8. Representative LC-MS chromatogram of the trypsin digest
S9. Representative LC-MS chromatogram of the chymotrypsin digest
S10. Representative LC-MS chromatogram of the Krakatoa digest
S11. Representative LC-MS chromatogram of the Vesuvius digest
S12. Peptide length distribution per protease
S13. Peptide charge distribution per protease
S14. Distribution of charges with respect to peptide length
S15. Redundancy in sequence coverage by unique peptide reads for triplicate
S16. Redundant reads per amino acid for one replicate
S17. CDR regions coverage by HTA proteases
S18. Unique peptides spanning CDR1 and CDR2 and the full CDR3 region
S19. Redundant reads per amino acid as obtained by de novo sequencing
S20. Unique reads per protease
S21. *De novo* alignment mistakes for the variable regions of all four antibodies
S22. EAciD outperforms CID on non-tryptic digests for *de novo* sequencing

Tables

S1. Sequences of the heavy and light chains of the antibodies used in the four monoclonal antibody mixture
S2. Summary of unique peptides, PSMs, and MS2 scans observed in each combination of protease and MS fragmentation method

Supplementary Figures

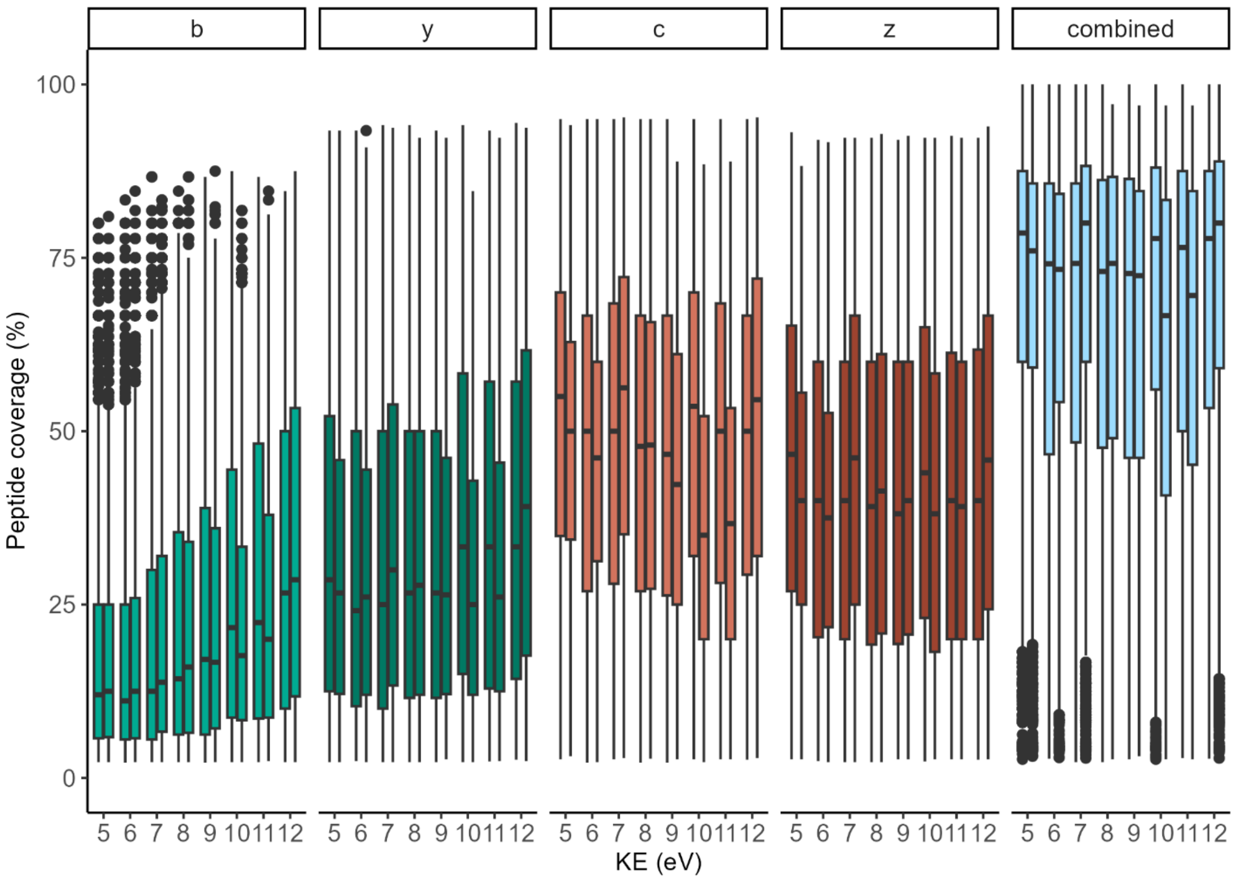

Figure S1. Optimization of kinetic energy (KE) parameters for the EAciD method. The figure depicts the median peptide sequence coverages (y-axis) by ion series (b-, y-, z-, and c-ions) and the combined coverage originating from a Krakatoa digest. The data were collected during optimization of the EAD method using various kinetic energies ranging from 5 to 12 eV (x-axes). As kinetic energy increases, the proportion of b-ions in the EAD fragmentation also rises, indicating enhanced secondary fragmentation and greater occurrence of neutral losses. We chose 9 eV as an intermediate kinetic energy setting to complement the supplemental collisional activation for the hybrid EAciD approach. The data originates from two replicate runs acquired at each kinetic energy setting.

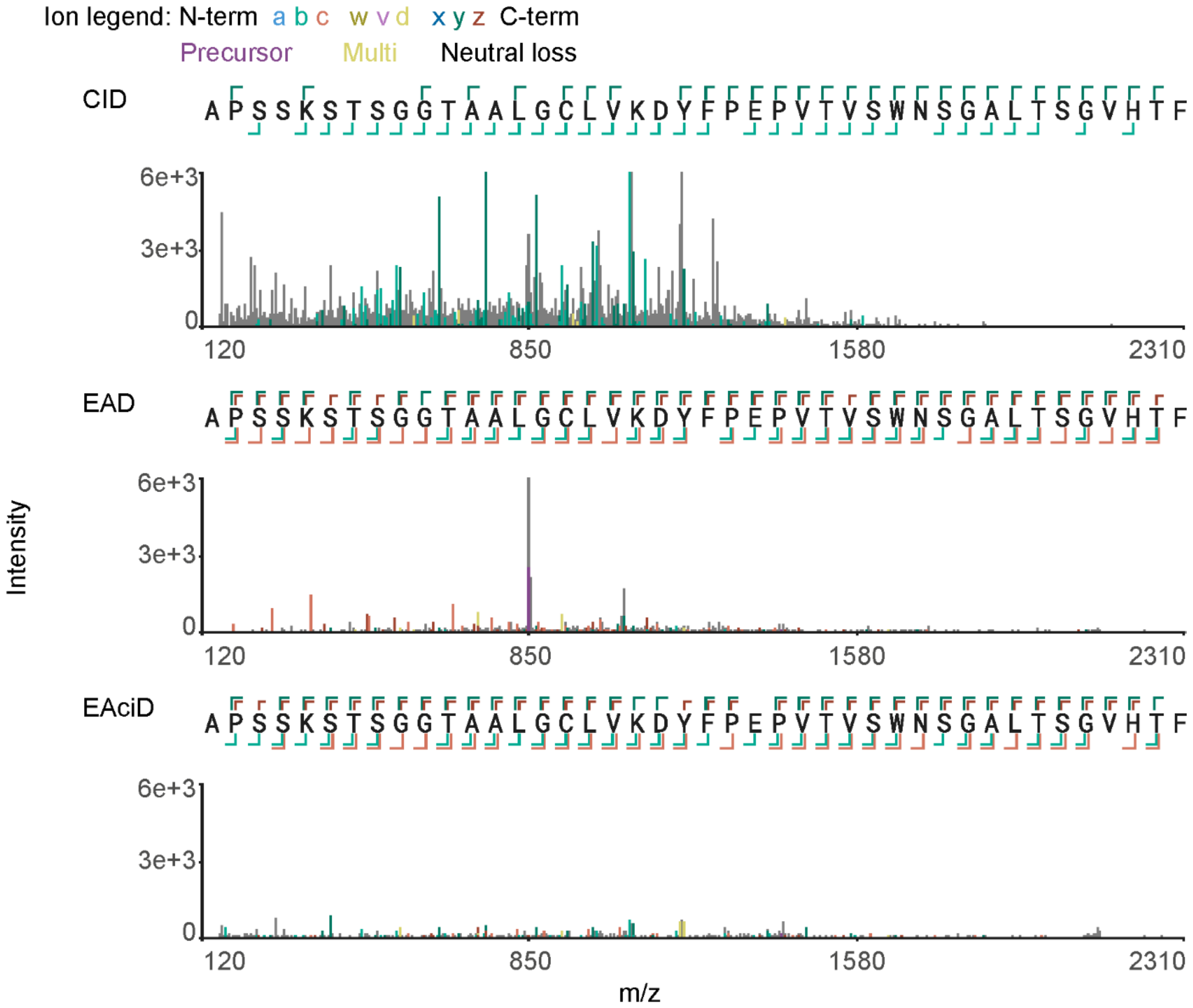

Figure S2. Intensity-normalized representative fragmentation spectra generated with CID, EAD and EAciD. The spectra correspond to a 42 amino acid long peptide, originating from the TZB heavy chain, obtained through digestion with Krakatoa. For comparison purposes, the intensity on the y-axis has been adjusted to 6e3 intensity and only a-, b-, c, x-, y-, and z- ions have been annotated. The cartoon at the top of each spectrum shows the amino acid sequence, whereby always multiple fragment ions (e.g. b-, c-, y-, z-) cover each amino acid throughout the entire peptide sequence.

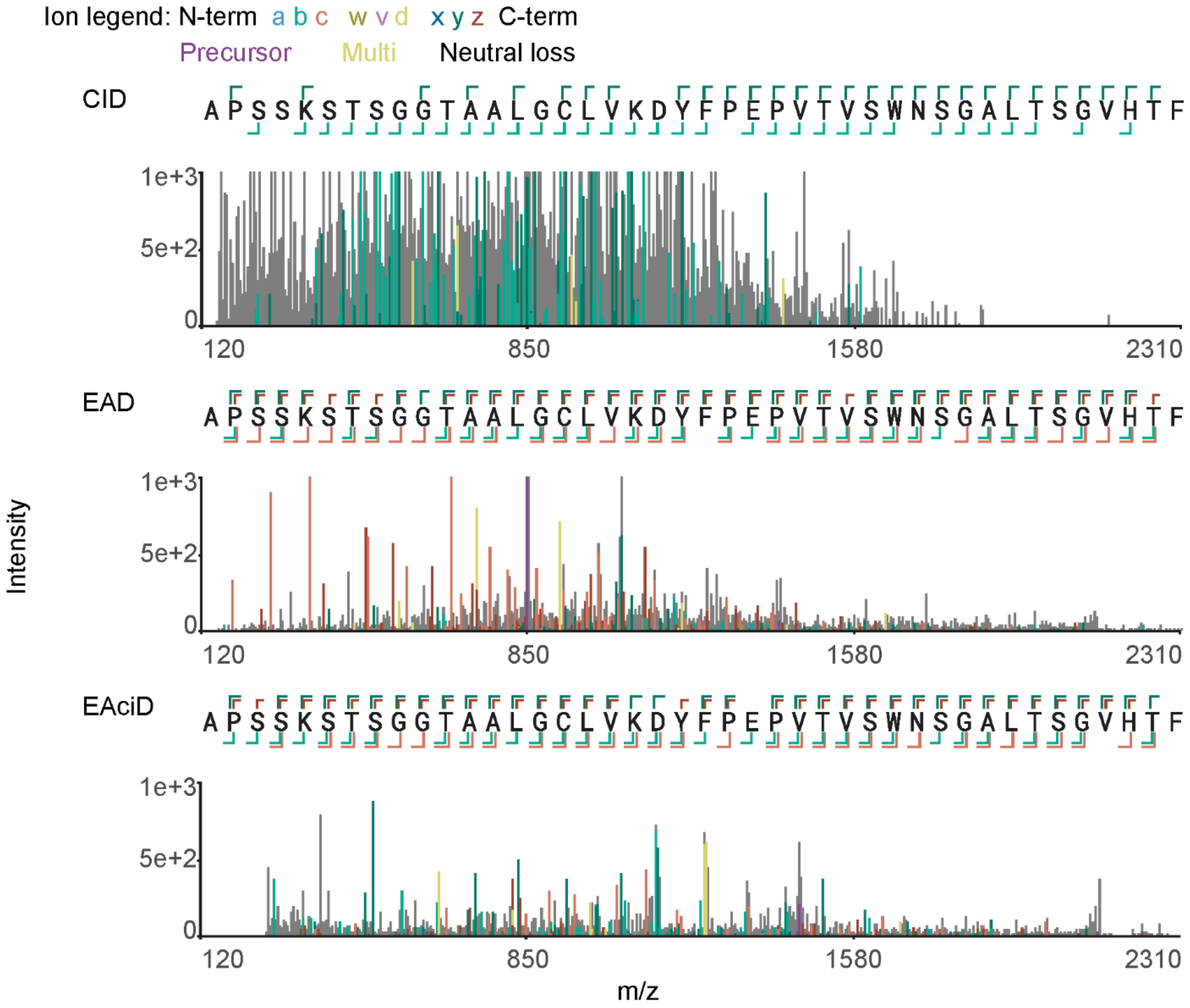

Figure S3. Magnified representative fragmentation spectra generated with CID, EAD and EAciD. The spectra correspond to a 42 amino acid long peptide, originating from the TZB heavy chain, obtained through digestion with Krakatoa. The intensity on the y-axis has been zoomed to 1e3 intensity in all cases for comparison purposes. The cartoon at the top of each spectrum shows the amino acid sequence, whereby always multiple fragment ions (e.g. b-, c-, y-, z-) cover each amino acid throughout the entire peptide sequence.

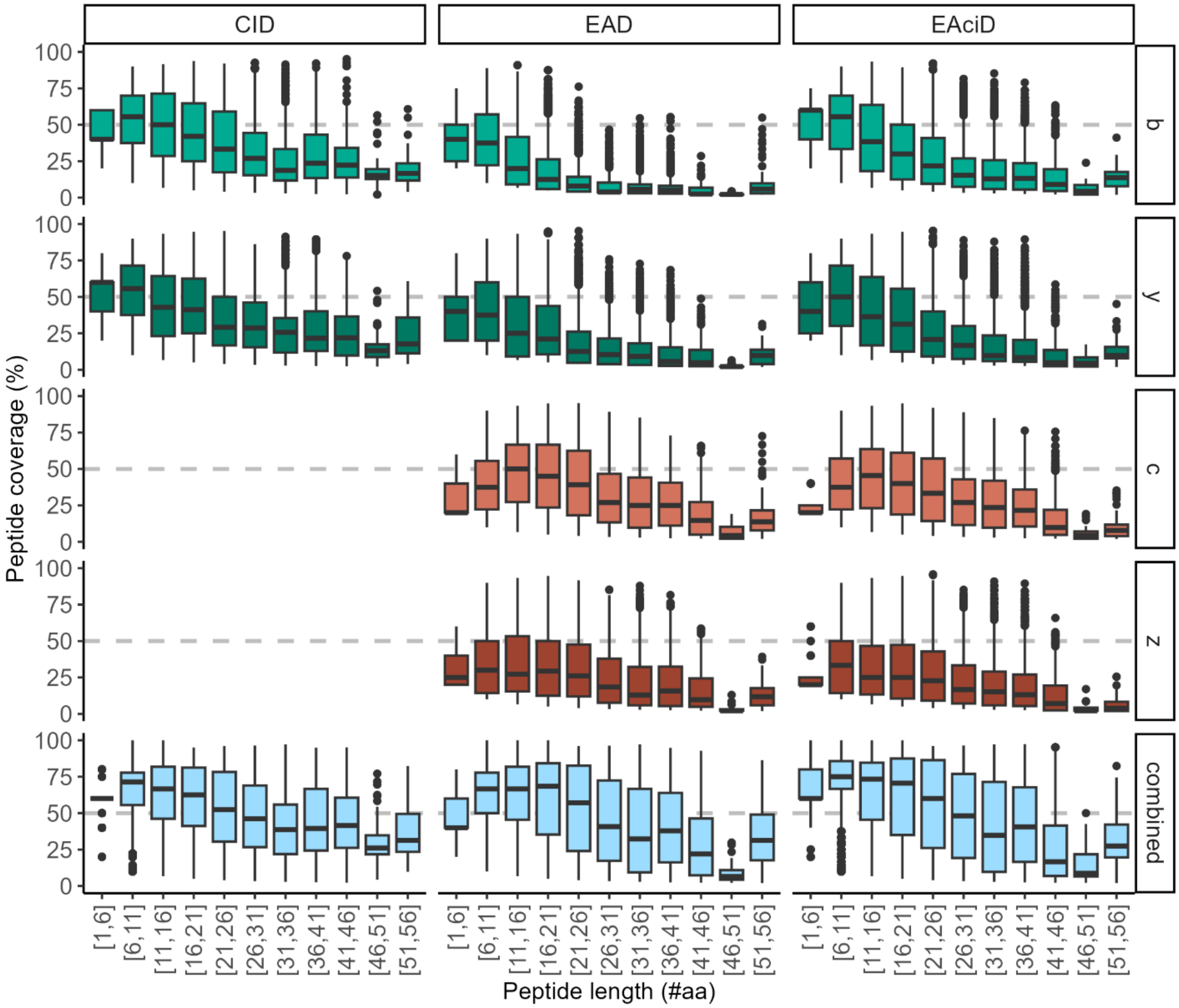

Figure S4. EAciD performs especially well on long peptide reads. Peptide sequence coverage across different peptide ion lengths for the fragmentation methods tested. Results show that CID yielded primarily b- and y- ion series, while c- and z- ion series are prevalent in EAD. The hybrid use of these two fragmentation methods, EAciD, provides good peptide coverage for all b-, y-, c- and z- ion series. Of note, the presence of b- and y-ions in pure EAD fragmentation indicates secondary fragmentation and neutral losses.[1–3]

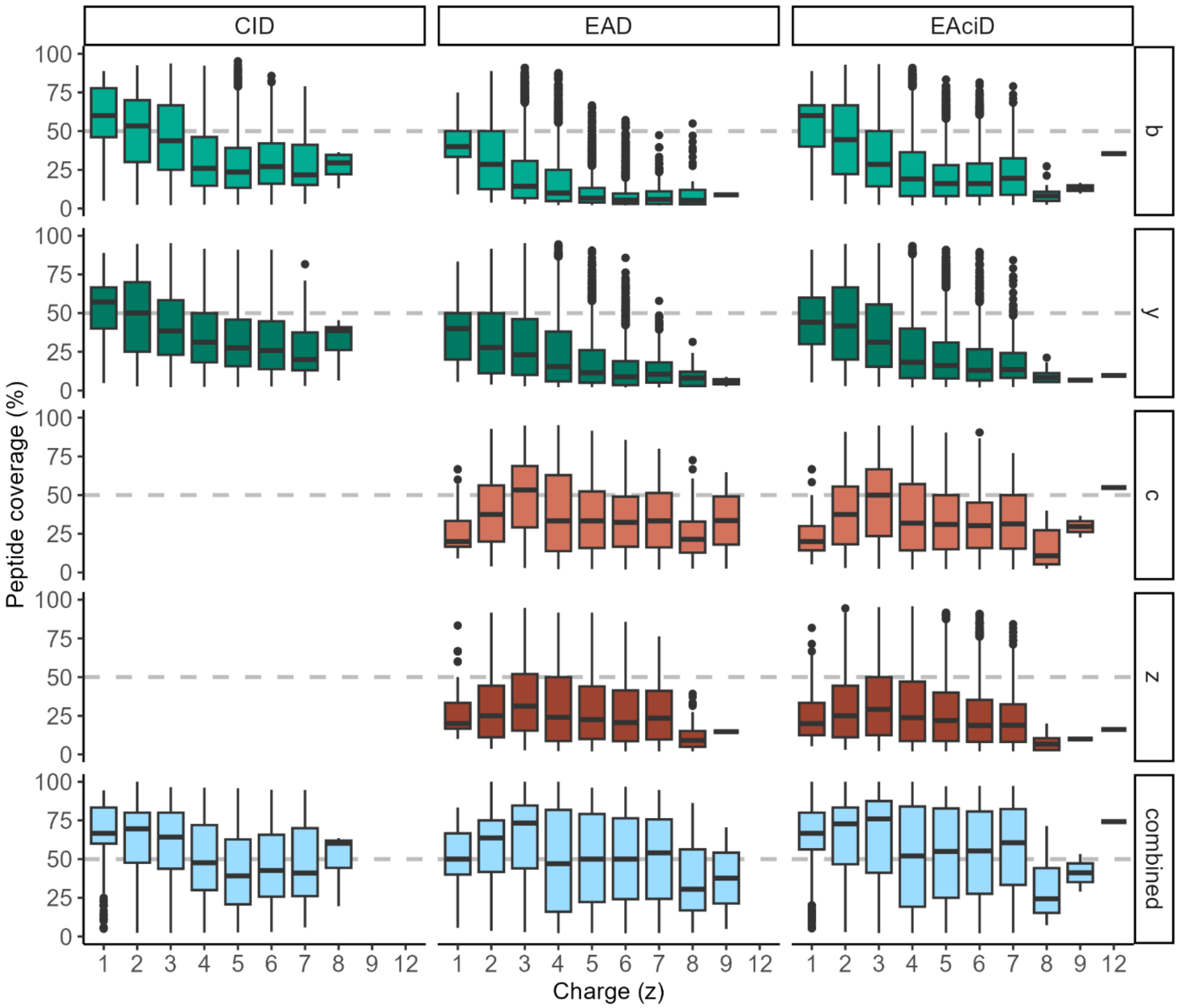

Figure S5. EAciD provides high peptide coverage across all peptide charges. Peptide coverage for peptide ion precursors in/with different charge states for the fragmentation methods tested. Results show that EAciD was able to generate more consistency in the peptide coverage for all ion types across the different charges, especially in the case of c- and z-ions.

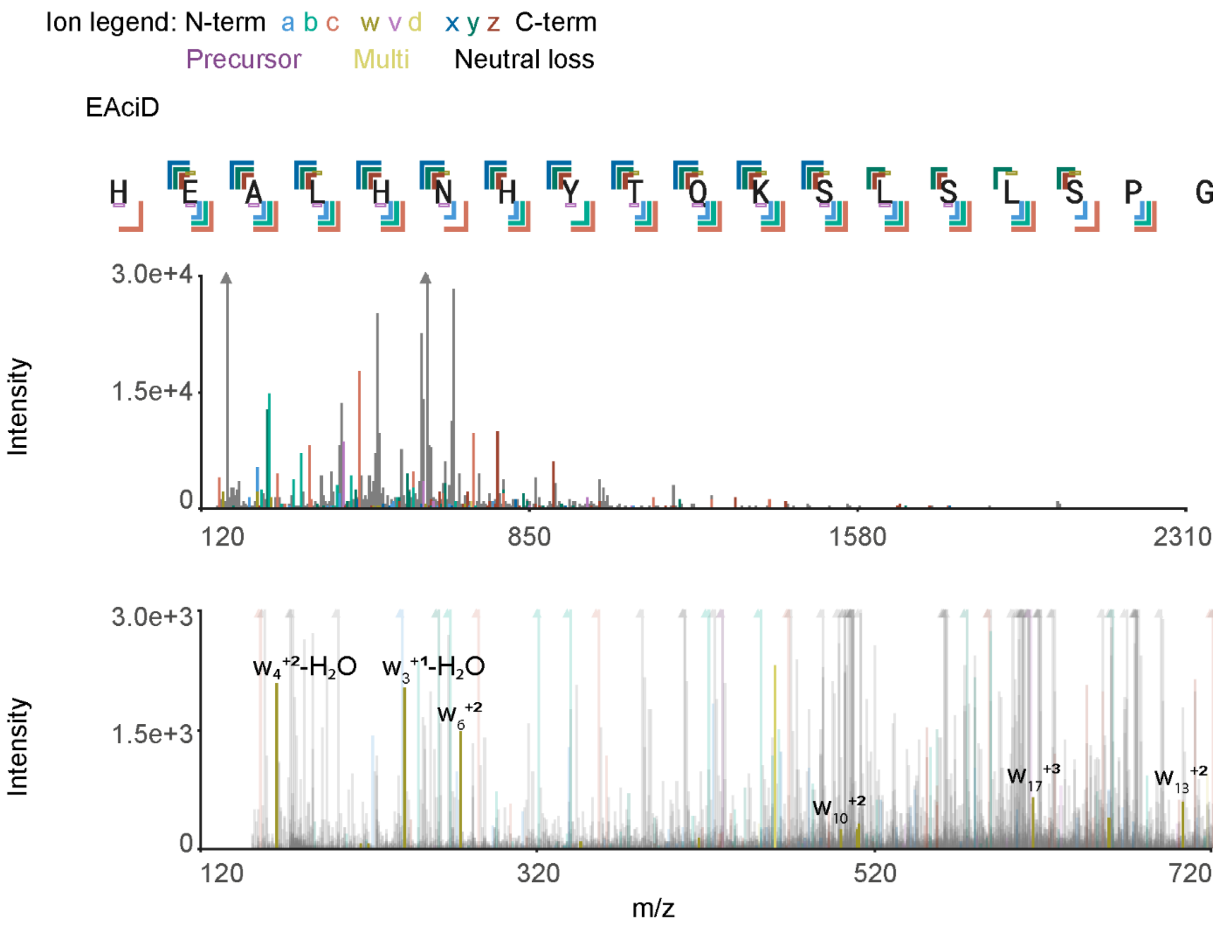

Figure S6. Representative fragmentation spectrum highlighting w- ions originating from Leucine. The spectra correspond to a peptide obtained through digestion with Vesuvius. The upper spectrum shows all annotated fragment ions (e.g. b-, c-, y-, z-) covering the amino acid positions of the peptide. The cartoon at the top of the upper spectrum shows the amino acid sequence of the peptide. The bottom spectrum shows the zoomed-in specific w-ions of the same peptide originating from the amino acid Leucine.

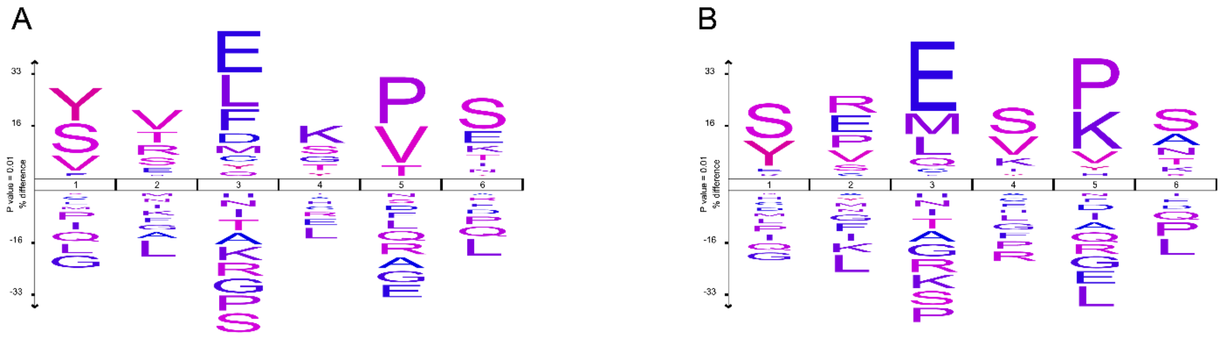

Figure S7. Sequence logos for (A) Krakatoa and (B) Vesuvius protease cleavage specificities. The specificities were determined for the data generated from the four mAb mixture analyzed by EAciD method. The logos were generated with IceLogo[4] against the human reference proteome with a 0.01 P-value cutoff. The cleavage site is localized C-terminal of the 3rd position. The observed cleavage specificities are similar to previously published data.[5,6] The minor differences in the cleavage specificities, when compared to previous reports, arise most likely from using here a simple sample consisting of just four alike mAbs.

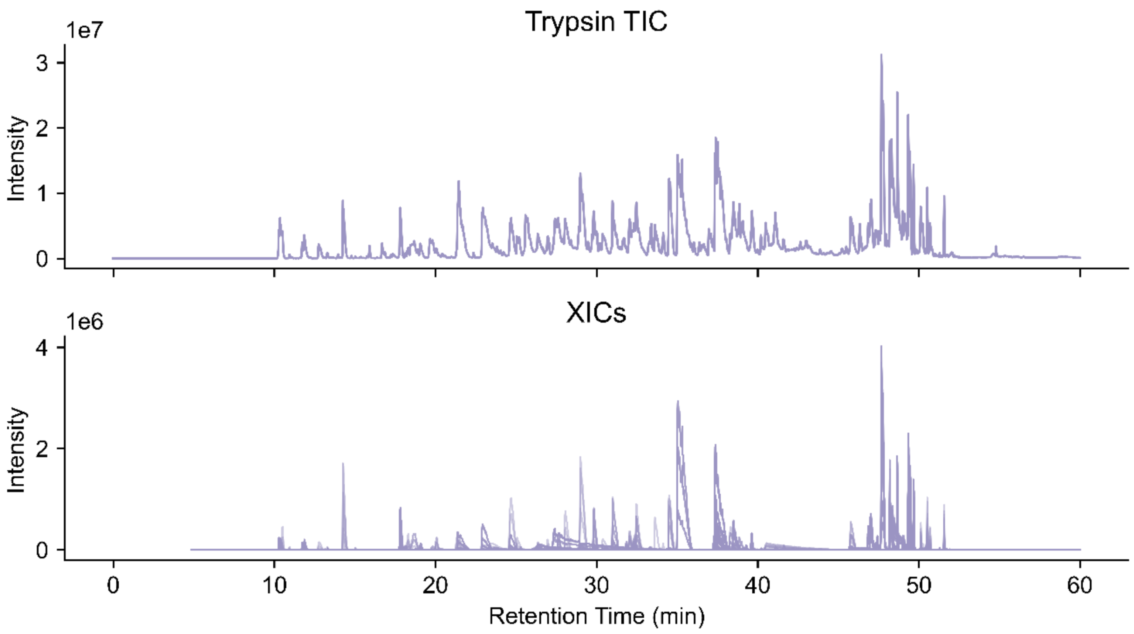

Figure S8. Representative LC-MS chromatogram of the trypsin digest. Total ion chromatogram (TIC) of the trypsin digest (top) and extracted ion chromatograms (XICs) of the Byonic-identified peptides from the tryptic digest (bottom).

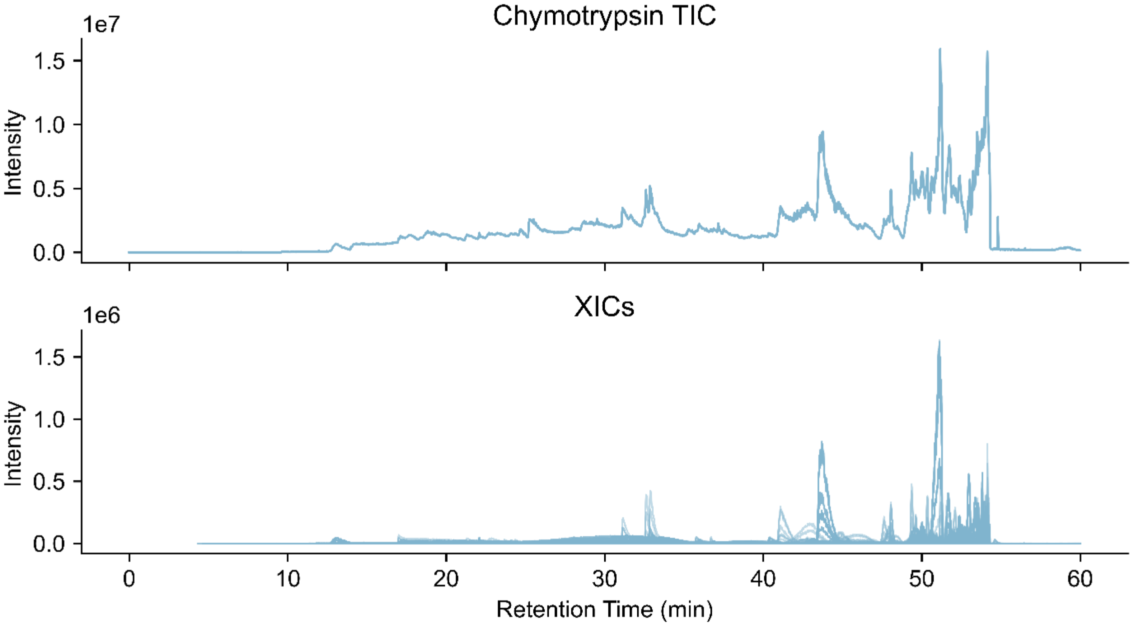

Figure S9. Representative LC-MS chromatogram of the chymotrypsin digest. Total ion chromatogram (TIC) of the chymotrypsin digest (top) and extracted ion chromatograms (XICs) of the Byonic-identified peptides from the chymotryptic digest (bottom).

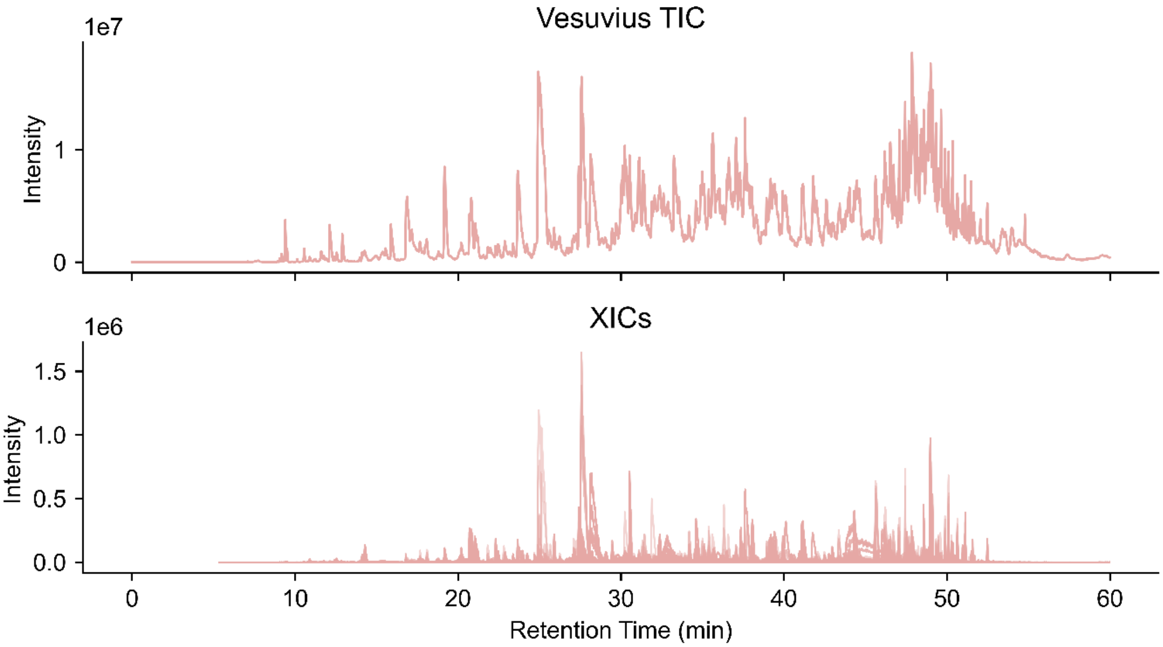

Figure S10. Representative LC-MS chromatogram of the Vesuvius digest. Total ion chromatogram (TIC) of the Vesuvius digest (top) and extracted ion chromatograms (XICs) of the Byonic-identified peptides from the Vesuvius digest (bottom).

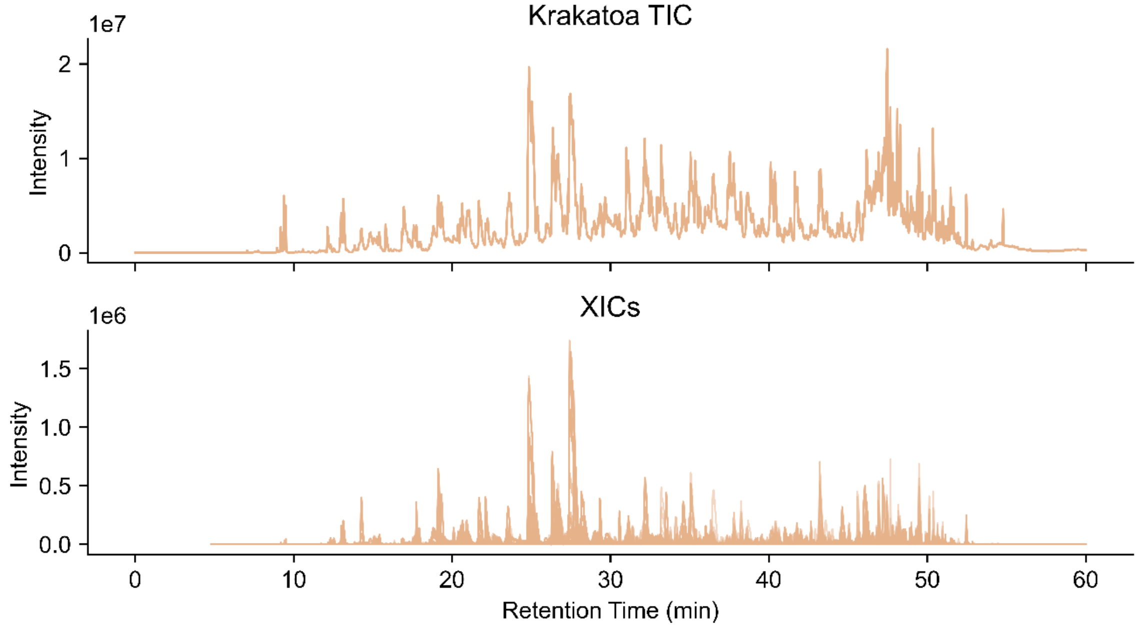

Figure S11. Representative LC-MS chromatogram of the Krakatoa digest. Total ion chromatogram (TIC) of the Krakatoa digest (top) and extracted ion chromatograms (XICs) of the Byonic-identified peptides from the Krakatoa digest (bottom).

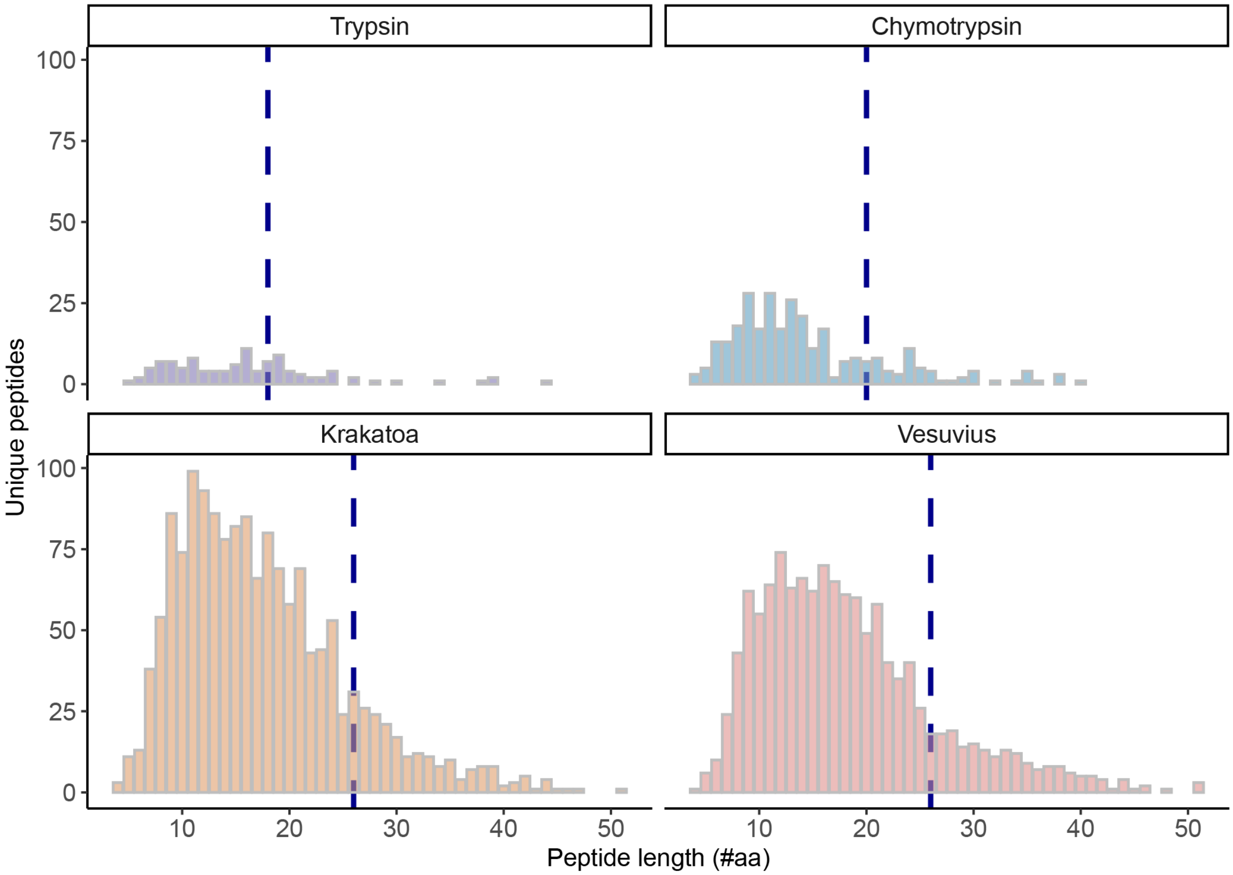

Figure S12. Peptide length distribution per protease. The bar plot depicts the peptide length distribution of the unique peptides identified per protease. Vesuvius and Krakatoa show a higher number of unique peptide identifications and a broader peptide length distribution, compared to trypsin and chymotrypsin. Remarkably, the HTA-proteases also produce a substantial number of longer peptides, resulting in a higher median peptide length, which is of great value in *de novo* sequencing to achieve better sequence coverage. The median ranges are 18, 20, 26 and 26 respectively for trypsin, chymotrypsin, Vesuvius, and Krakatoa, as indicated by the blue line. This data originates solely from the data generated in EAciD mode, filtered for Byonic score ≥150 and log ≥ 3 from n= 3 technical replicates.

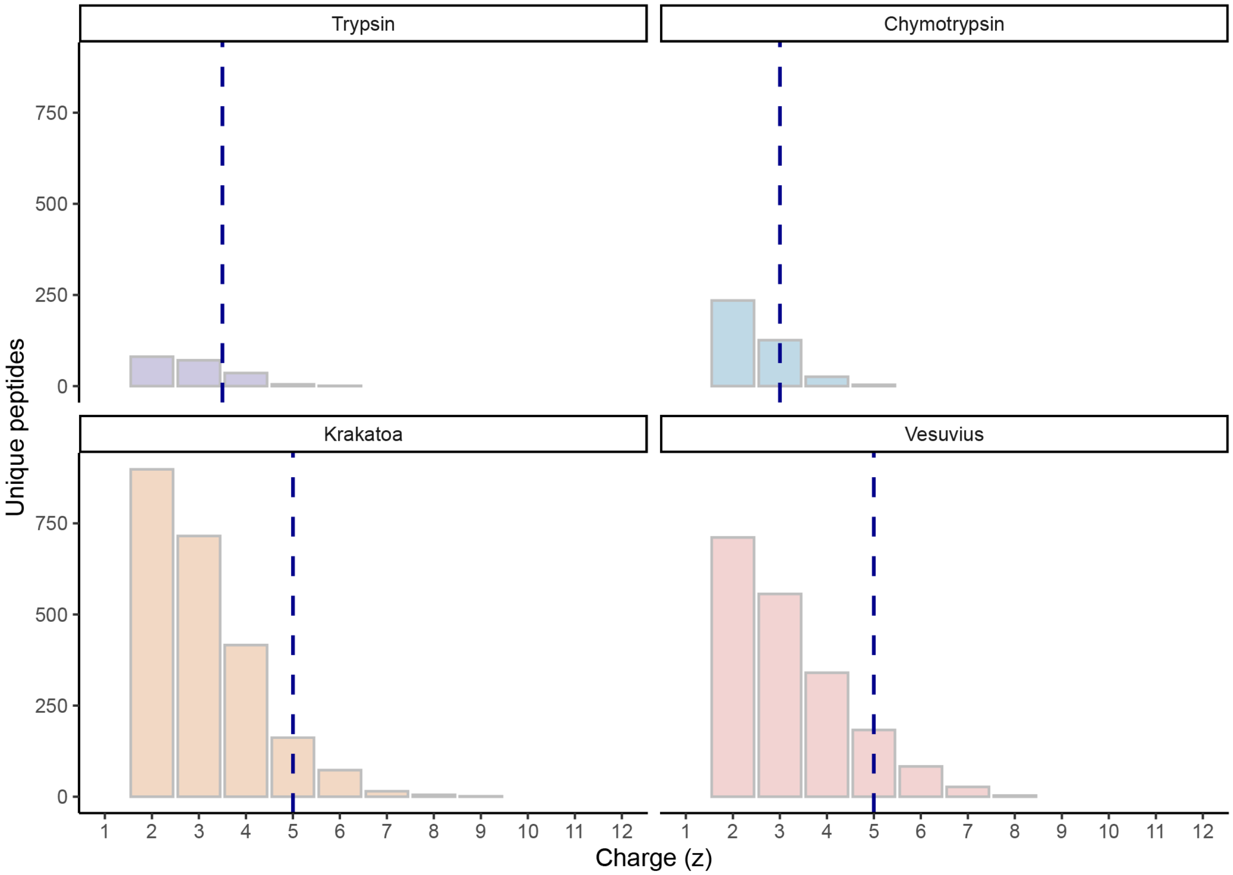

Figure S13. Peptide charge distribution per protease. The bar plot depicts the charge distribution of the unique peptides identified per protease. Vesuvius and Krakatoa show a higher number of unique peptide identifications and a considerably broader charge distribution, compared to trypsin and chymotrypsin. The median ranges are +3.5, +3.0, +5.0, and +5.0, respectively, for trypsin, chymotrypsin, Vesuvius, and Krakatoa, as indicated by the blue line. This data originates solely from the data generated in EAciD mode, filtered for Byonic score ≥150 and log ≥ 3 from n= 3 technical replicates.

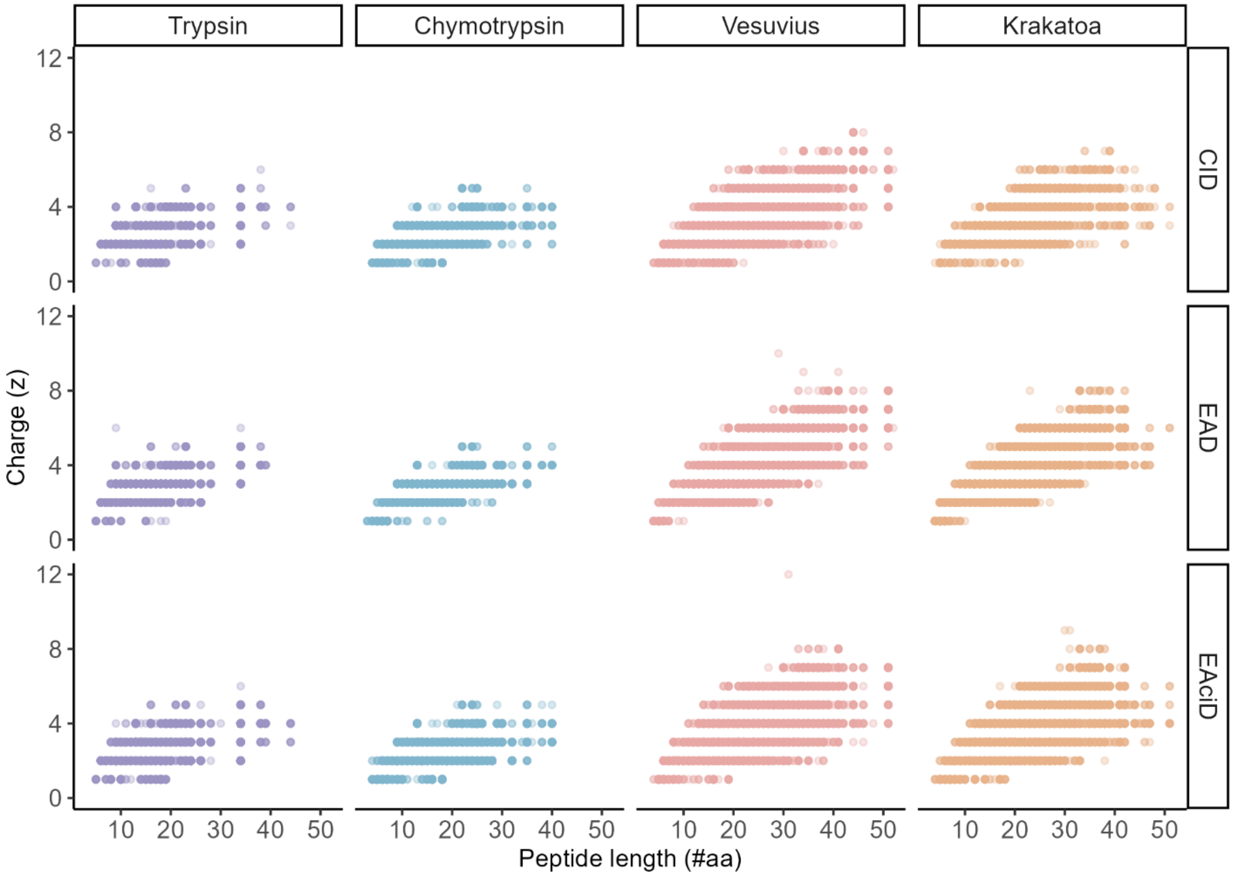

Figure S14. Distribution of charges with respect to peptide length. The data displayed represents the charge and peptide length distribution of the three replicates per protease and MS method. Vesuvius and Krakatoa show a broader charge and peptide length distribution compared to trypsin and chymotrypsin, which are limited to lower charge states and peptide length. This data was filtered for Byonic score ≥150 and log ≥ 3 and originates from n= 3 technical replicates.

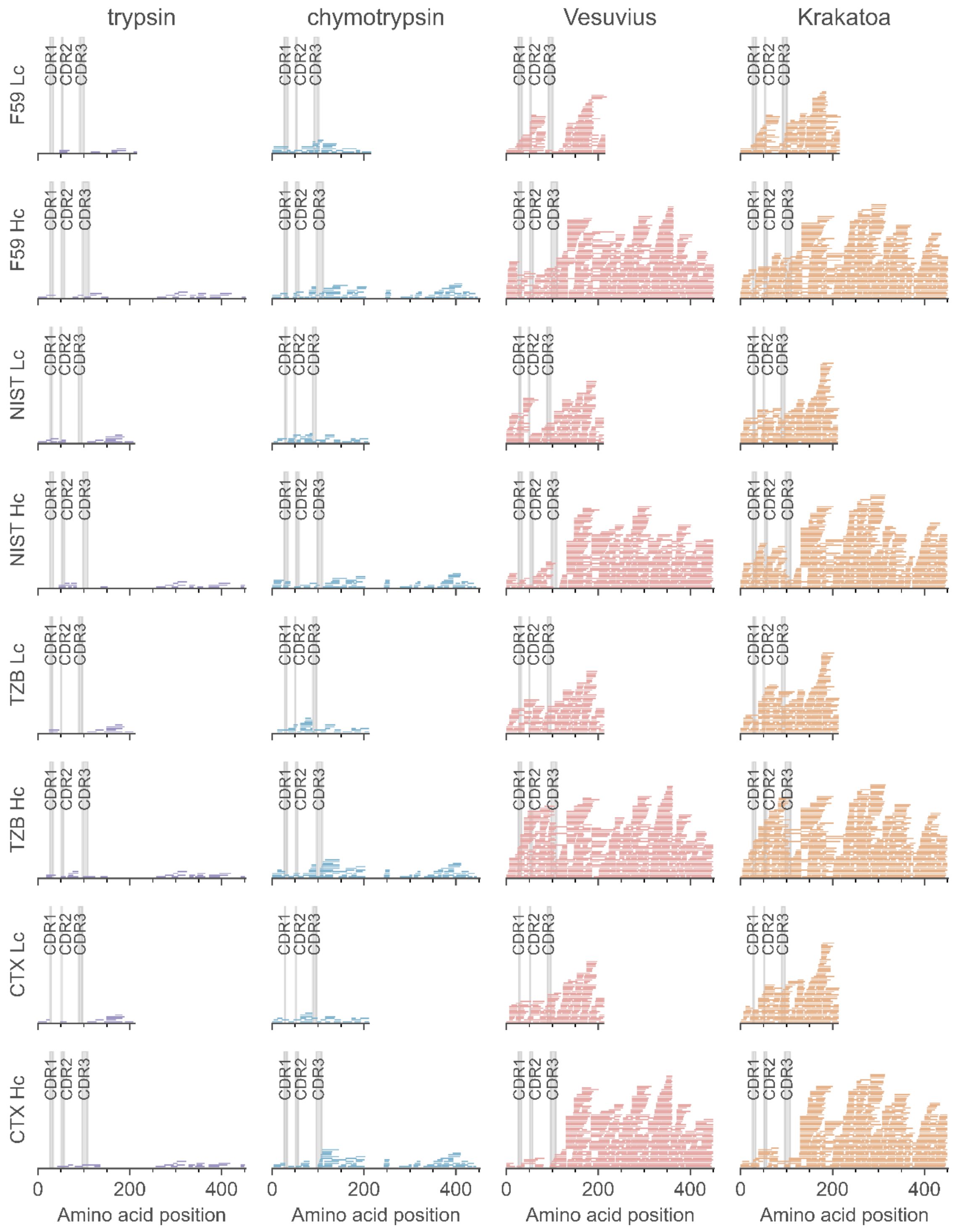

Figure S15. Redundancy in sequence coverage by unique peptide reads. Depicted are the unique peptides generated by trypsin (purple), chymotrypsin (blue), Vesuvius (red), and Krakatoa (orange) spanning over the Lc and Hc sequences of all four mAbs. Each unique peptide detected is visualized as a line covering the respective region in the protein sequence. CDR regions are highlighted in gray and labeled. This data originates solely from the data generated in EAciD mode, filtered for Byonic score ≥150 and log ≥ 3 from n= 3 technical replicates.

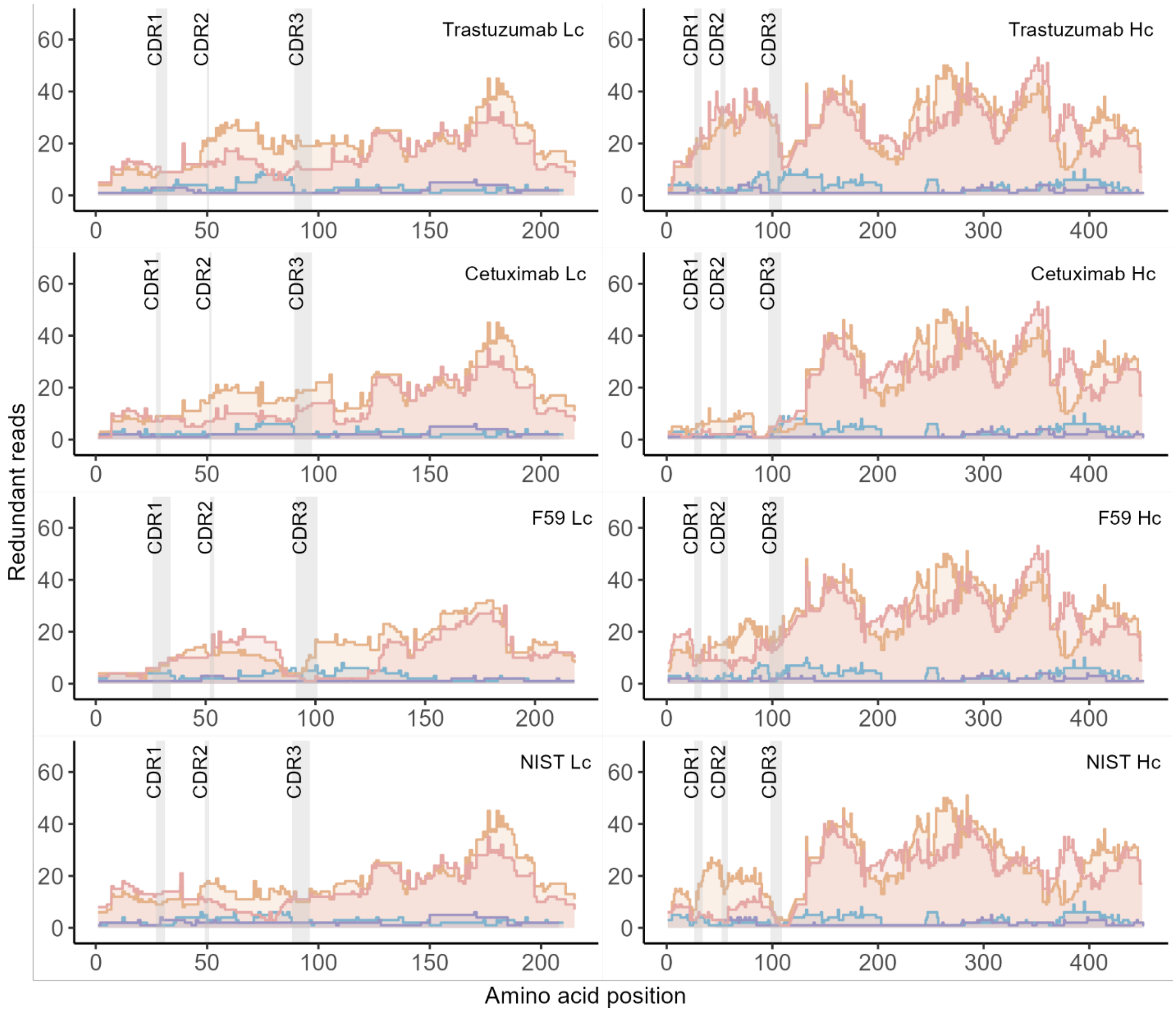

Figure S16. Redundant reads per amino acid for one replicate. Each graph depicts the number of times each amino acid in the sequence of one of the antibody chains is covered by unique peptides in one replicate. The data displayed originates from the digest by trypsin (purple), chymotrypsin (blue), Vesuvius (pink), or Krakatoa (orange). The HTA-proteases, Vesuvius and Krakatoa, consistently provide substantially more sequence reads for all the light and heavy chains of the studied antibodies, including the CDR regions. The individual reads, including both shorter and longer reads, are depicted.

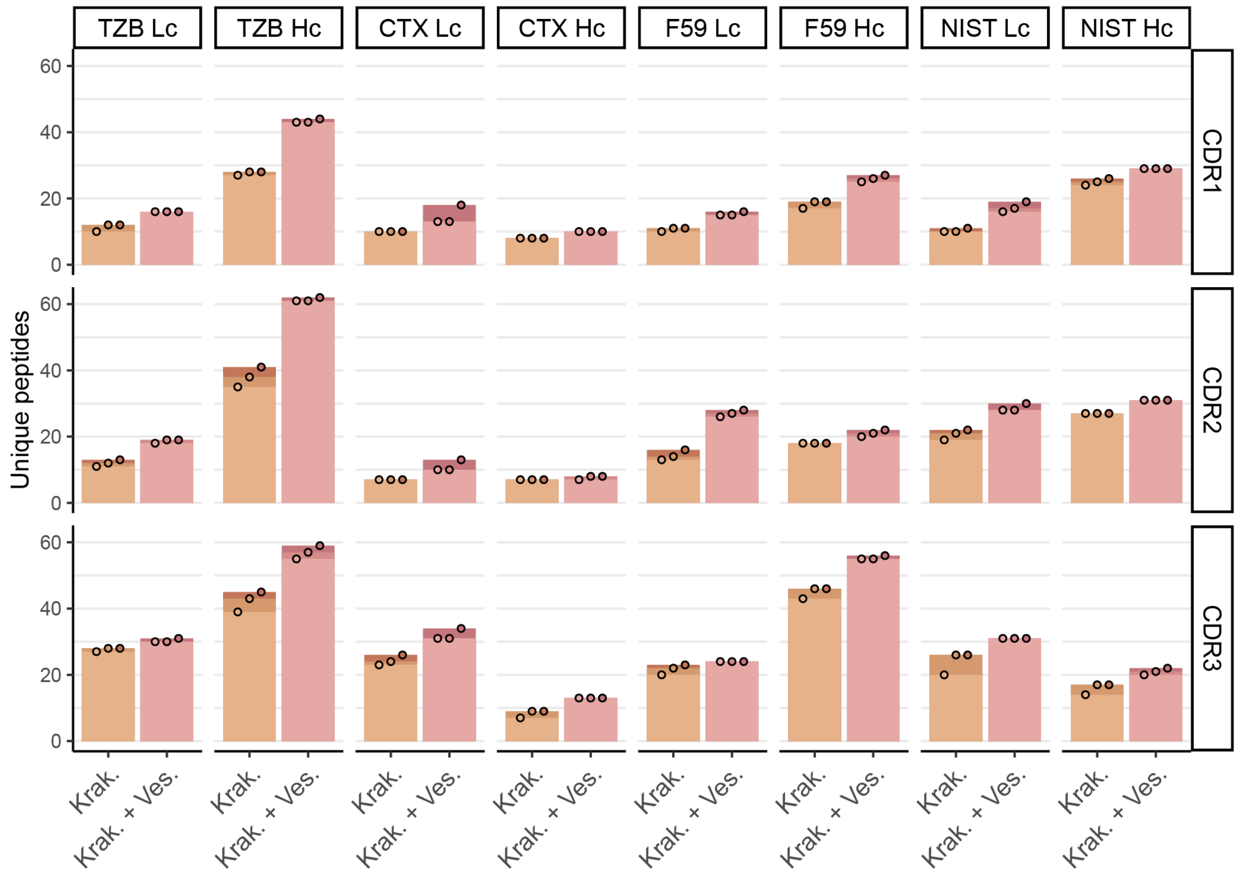

Figure S17. CDR regions coverage by HTA proteases. The sum of unique peptides is shown covering the CDR regions per individual antibody, either by using Krakatoa or by combining data from the Krakatoa and Vesuvius digest. Although the combined usage of the two proteases marginally increases the number of identified unique peptides, data from a single Krakatoa digest are sufficient to fully cover all CDR regions. Each black circle represents the cumulative unique peptides detected per replicate. This data originates solely from the data generated in EAciD mode, filtered for Byonic score ≥150 and log ≥ 3 from n= 3 technical replicates.

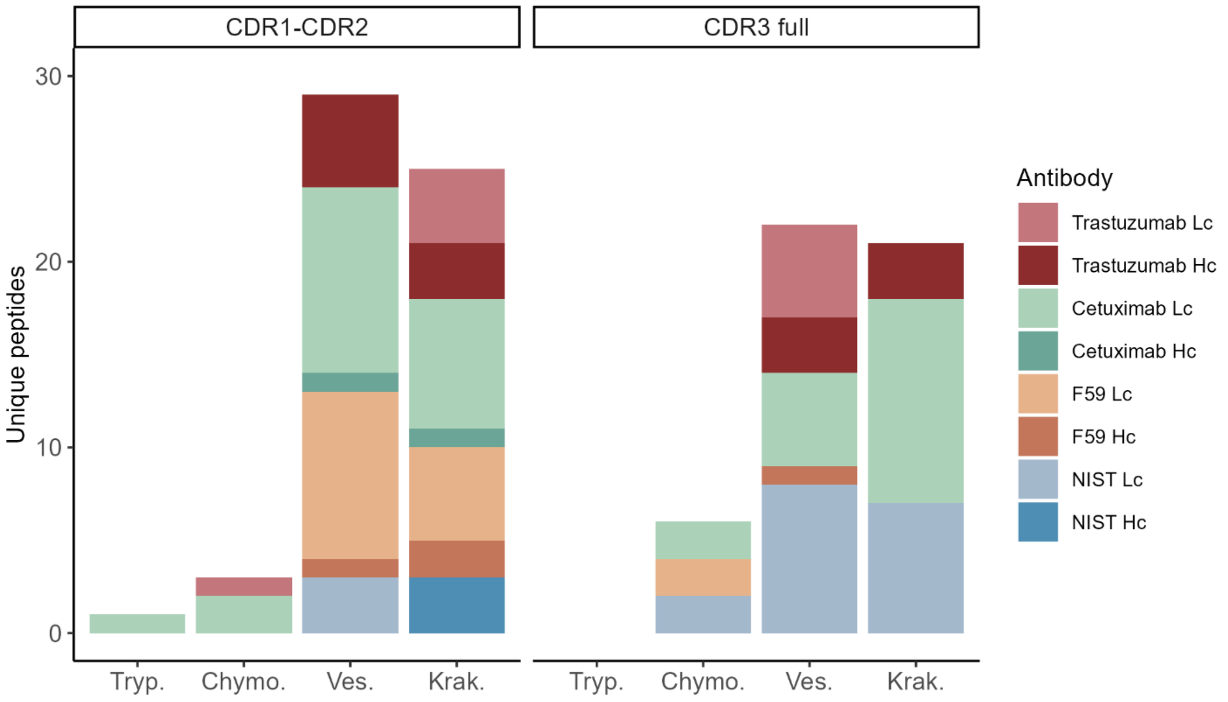

Figure S18. Unique peptides spanning CDR1 and CDR2 and the full CDR3 region. Unique peptides were mapped to the CDR regions of the corresponding mAb sequences; peptides covering parts of both CDR1 and CDR2 were labelled as "CDR1-CDR2" and peptides covering the full CDR3 region, including the previous amino acid in the sequence, were labeled as "CDR3 full". Vesuvius and Krakatoa provide the highest unique peptides covering these regions. Additionally, the combination of both HTA-proteases generates peptides for all four antibodies that span both CDR1 and CDR2.

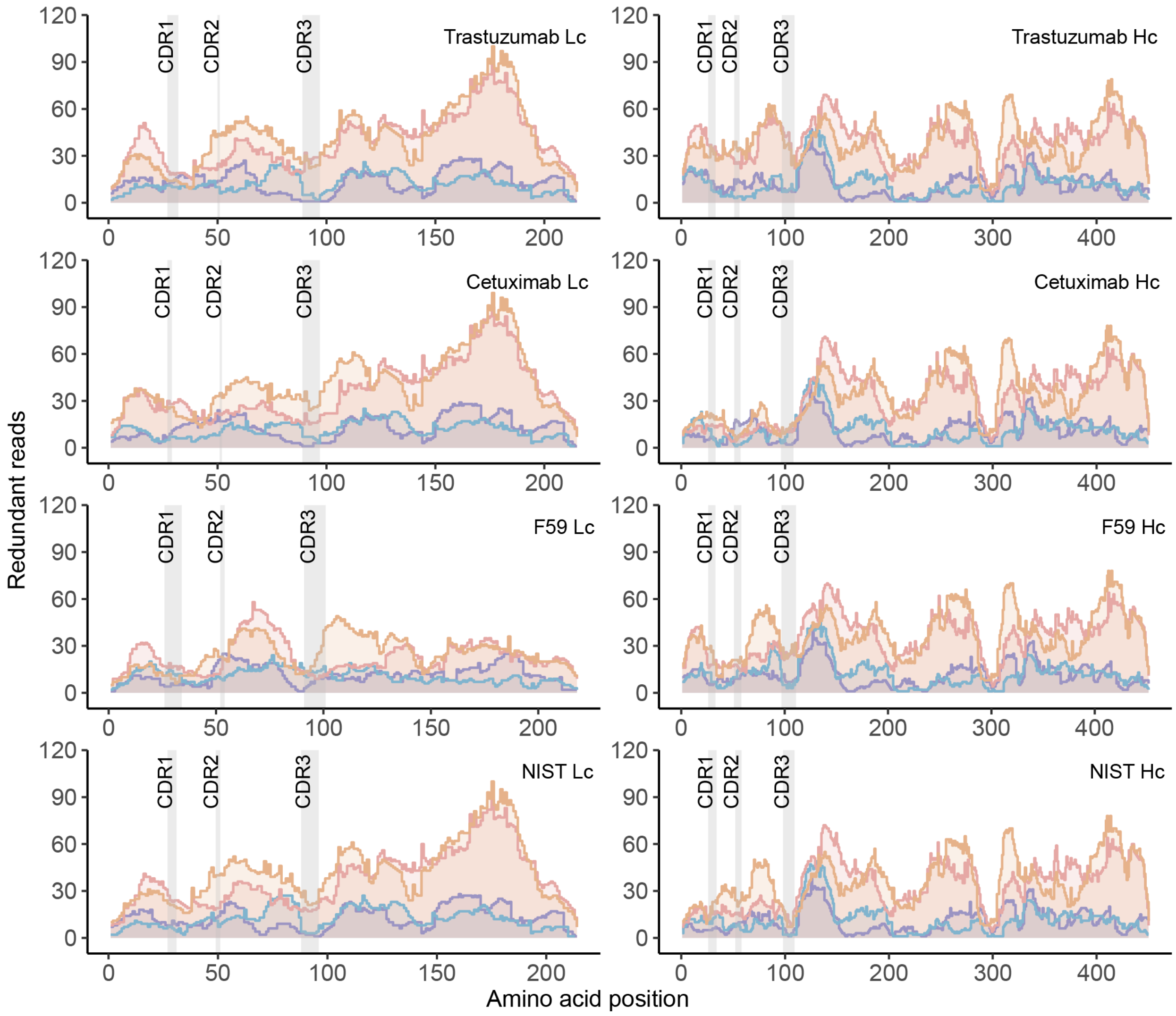

Figure S19. Redundant reads per amino acid as obtained by *de novo* sequencing. Each graph depicts the number of times each amino acid in the sequence of one of the antibody chains is covered by unique de novo annotated peptides. The data displayed originates from the digest by trypsin (purple), chymotrypsin (blue), Vesuvius (pink), or Krakatoa (orange). The HTA-proteases, Vesuvius and Krakatoa, consistently provide substantially more *de novo* sequence reads for all the light and heavy chains of the studied antibodies, including the CDR regions. The individual reads, including both shorter and longer reads, are depicted. This data originates solely from the data generated in EAciD mode, filtered for PEAKS ALC score ≥80, and for Stitch Cutoff Score 8 and Enforce Unique 0.8 for Template Matching, from n= 3 technical replicates.

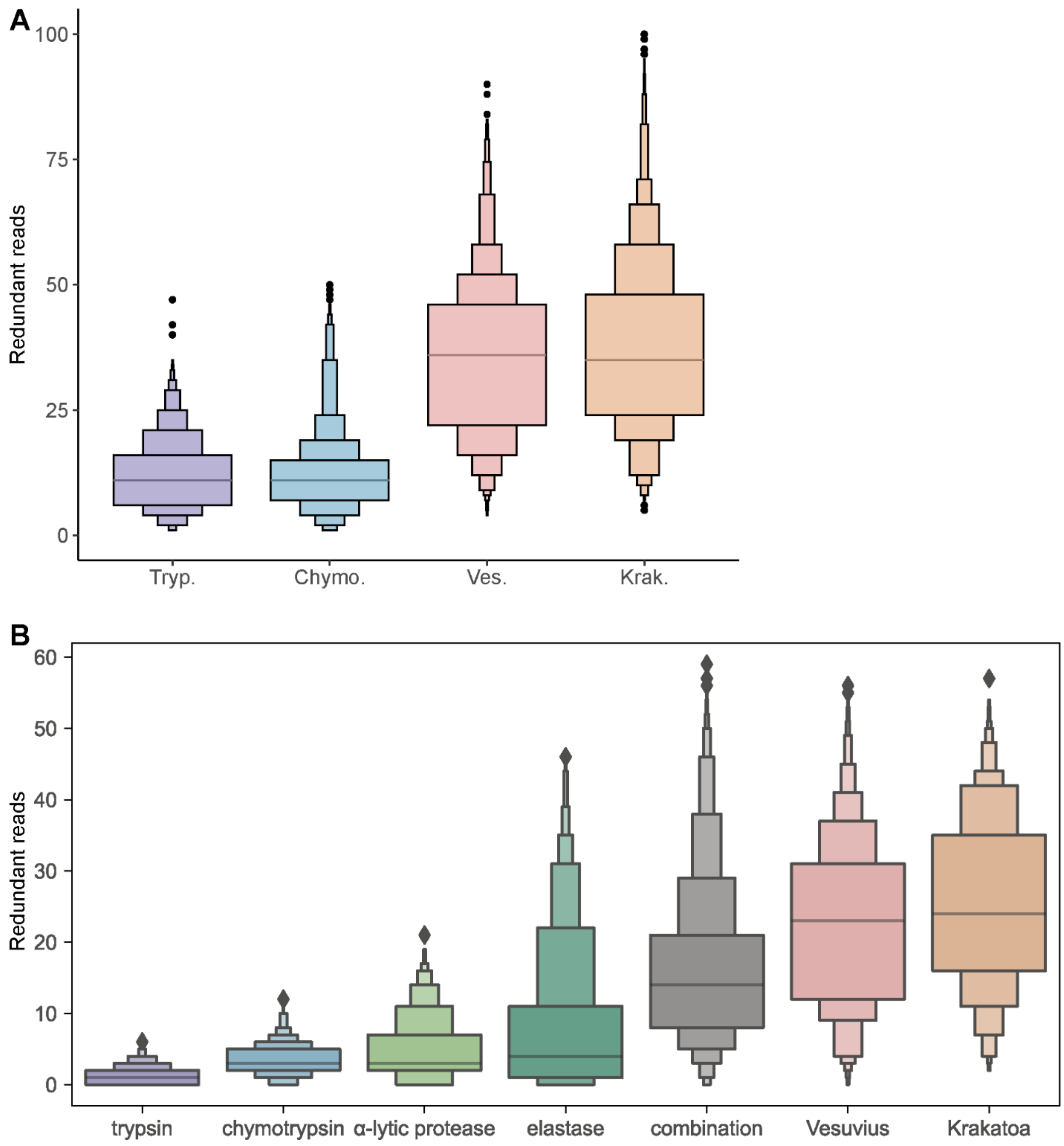

Figure S20. Unique reads per protease. (A) Unique *de novo* reads per protease. Cumulative data from both the Lc and Hc chains of all four investigated antibodies. *De novo* unique reads were defined based on the amino acid position on the sequences; all peptides that, based on Stitch annotation, did not start with insertions or deletions compared to the consensus sequence were included. The median number of redundant reads corresponds to 11, 11, 36, and 35 for trypsin, chymotrypsin, Vesuvius, and Krakatoa, respectively. The plots represent results from n=3 replicates. (B) Distribution of the unique redundant sequence reads per protease. Cumulative results from a triplicate analysis of database matching search in Byonic combined visualized for Lc and Hc chains of all four investigated antibodies. The "combination" corresponds to all unique reads detected by trypsin, chymotrypsin, elastase, and α-lytic protease. The median number of redundant sequence reads is 1, 3, 3, 4, 14, 23, and 24 for trypsin (purple), chymotrypsin (blue), α-lytic protease (light green), elastase (dark green), combination (grey), Vesuvius (red), and Krakatoa (orange), respectively. The elastase and α-lytic protease results were analyzed and filtered identically as the HTA-proteases data.

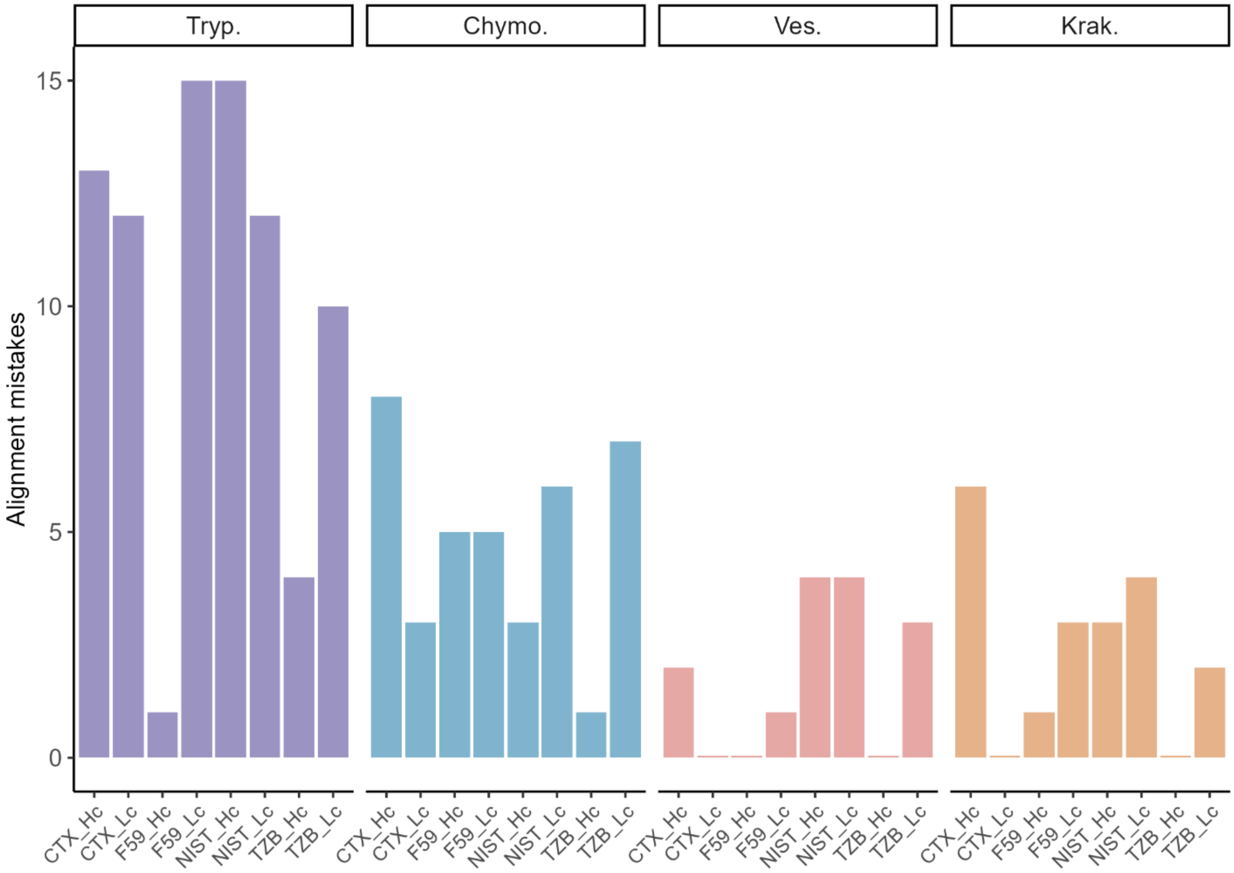

Figure S21. ***De novo*** **alignment mistakes for the variable regions of all four antibodies.** Data represents the number of mistakes generated when matching the template to the consensus sequence. No alignment mistakes were detected for CTX Lc and TZB Hc with Vesuvius and Krakatoa, and for F59 Hc with Vesuvius. The alignment mistakes were calculated for results from n=3 replicates.

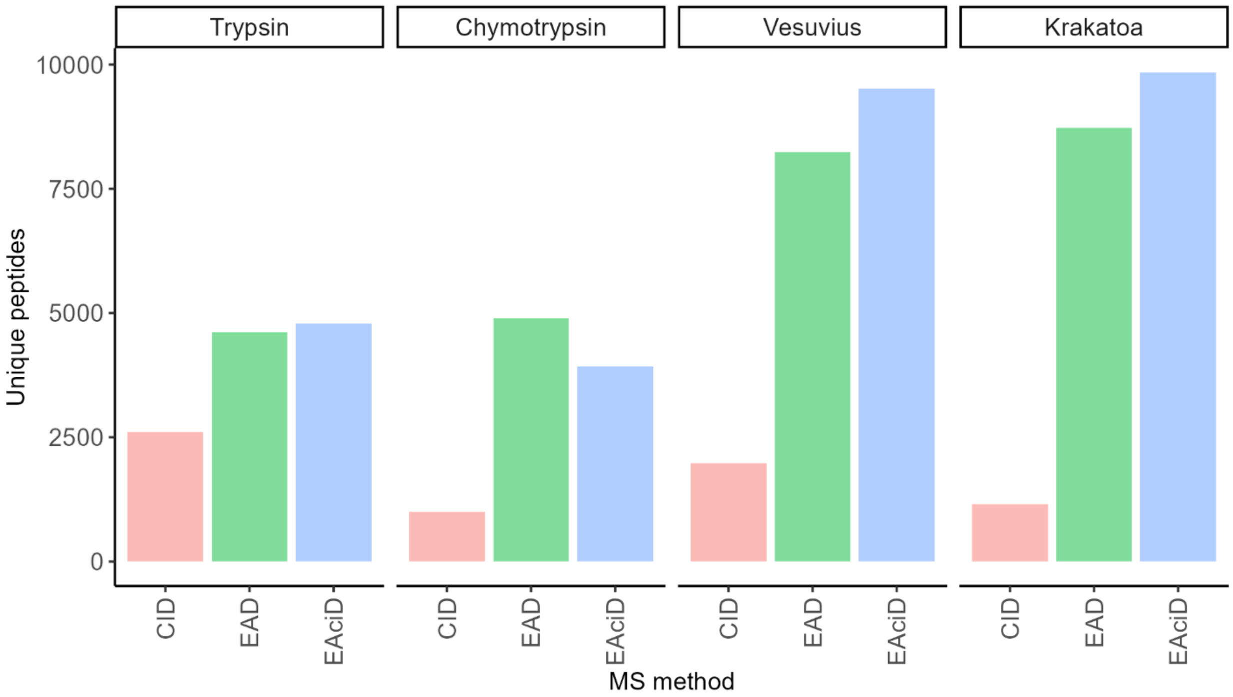

Figure S22. **EAciD outperforms CID on non-tryptic digests for *de novo* sequencing**. *De novo* unique peptides detected across three replicates with PEAKS score ≥ 80. EAciD shows a notably superior performance for non-tryptic digests, substantially increasing the number of unique peptides detected compared to CID.

Supplementary Tables

Table S1. Sequences of the heavy and light chains of the antibodies used in the four monoclonal antibody mixture.

Description	Sequence
Trastuzumab<br>(Light chain)	DIQMTQSPSSLSASVGDRVTITCRASQDVNTAVAWYQQKPGKAPKLLIYSASFLYSGVPSRFSGSRSGTDFTL<br>TISSLQPEDFATYYCQQHYTTPPTFGQGTKVEIKRTVAAPSVFIFPPSDEQLKSGTASVVCLLNNFYPREAKVQ<br>WKVDNALQSGNSQESVTEQDSKDSTYSLSSTLTLSKADYEKHKVYACEVTHQGLSSPVTKSFNRGEC
Trastuzumab<br>(Heavy chain)	EVQLVESGGGLVQPGGSLRLSCAASGFNIKDTYIHWVRQAPGKGLEWVARIYPTNGYTRYADSVKGRFTISA<br>DTSKNTAYLQMNSLRAEDTAVYYCSRWGGDGFYAMDYWGQGTLVTVSSASTKGPSVFPLAPSSKSTSGGT<br>AALGCLVKDYFPEPVTVSWNSGALTSGVHTFPAVLQSSGLYSLSSVVTVPSSSLGTQTYICNVNHKPSNTKVD<br>KKVEPKSCDKTHTCPPCPAPELLGGPSVFLFPPKPKDTLMISRTPEVTCVVVDVSHEDPEVKFNWYVDGVEV<br>HNAKTKPREEQYNSTYRVVSVLTVLHQDWLNGKEYKCKVSNKALPAPIEKTISKAKGQPREPQVYTLPPSREE<br>MTKNQVSLTCLVKGFYPSDIAVEWESNGQPENNYKTTPPVLDSDGSFFLYSKLTVDKSRWQQGNVFSCSVM<br>HEALHNHYTQKSLSLSPG
Cetuximab<br>(Light chain)	DILLTQSPVILSVSPGERVSFSCRASQSIGTNIHWYQQRTNGSPRLLIKYASESISGIPSRFSGSGSGTDFTLSINS<br>VESEDIADYYCQQNNNWPTTFGAGTKLELKRTVAAPSVFIFPPSDEQLKSGTASVVCLLNNFYPREAKVQWK<br>VDNALQSGNSQESVTEQDSKDSTYSLSSTLTLSKADYEKHKVYACEVTHQGLSSPVTKSFNRGEC
Cetuximab<br>(Heavy chain)	QVQLKQSGPGLVQPSQSLSITCTVSGFSLTNYGVHWVRQSPGKGLEWLGVIWSGGNTDYNTPFTSRLSINK<br>DNSKSQVFFKMNSLQSNDTAIYYCARALTYYDYEFAYWGQGTLVTVSAASTKGPSVFPLAPSSKSTSGGTAAL<br>GCLVKDYFPEPVTVSWNSGALTSGVHTFPAVLQSSGLYSLSSVVTVPSSSLGTQTYICNVNHKPSNTKVDKRV<br>EPKSCDKTHTCPPCPAPELLGGPSVFLFPPKPKDTLMISRTPEVTCVVVDVSHEDPEVKFNWYVDGVEVHNA<br>KTKPREEQYNSTYRVVSVLTVLHQDWLNGKEYKCKVSNKALPAPIEKTISKAKGQPREPQVYTLPPSREEMTK<br>NQVSLTCLVKGFYPSDIAVEWESNGQPENNYKTTPPVLDSDGSFFLYSKLTVDKSRWQQGNVFSCSVMHEA<br>LHNHYTQKSLSLSPGK
F59<br>(Light chain)	QSALTQPASVSGSPGQSITISCTGTSSDVGGYNYVSWYQHHPGKAPKLLISEVSDRPSGVSSRFSGSKSGNTA<br>SLTISGLQAEDESMYFCSSYTDLTSFVVFGGGTKLTVLGQPKAAPSVTLFPPSSEELQANKATLVCLISDFYPGA<br>VTVAWKADSSPVKAGVETTTPSKQSNNKYAASSYLSLTPEQWKSHRSYSCQVTHEGSTVEKTVAPTECS
F59<br>(Heavy chain)	EPELVESGGGLAQPGTSLRLSCEASGFTFDDYAMHWVRQAPGRALEWVSGISWSSDNLAYSDSVEGRFTIS<br>RDNAKNSLYLQMNSLRLDDTAFYYCAKDVRPYYDFWAFDSWGRGTPVTVSSASTKGPSVFPLAPSSKSTSG<br>GTAALGCLVKDYFPEPVTVSWNSGALTSGVHTFPAVLQSSGLYSLSSVVTVPSSSLGTQTYICNVNHKPSNTK<br>VDKRVEPKSCDKTHTCPPCPAPELLGGPSVFLFPPKPKDTLMISRTPEVTCVVVDVSHEDPEVKFNWYVDGV<br>EVHNAKTKPREEQYNSTYRVVSVLTVLHQDWLNGKEYKCKVSNKALPAPIEKTISKAKGQPREPQVYTLPPSR<br>EEMTKNQVSLTCLVKGFYPSDIAVEWESNGQPENNYKTTPPVLDSDGSFFLYSKLTVDKSRWQQGNVFSCS<br>VMHEALHNHYTQKSLSLSPG
NIST<br>(Light chain)	DIQMTQSPSTLSASVGDRVTITCSASSRVGYMHWYQQKPGKAPKLLIYDTSKLASGVPSRFSGSGSGTEFTLTI<br>SSLQPDDFATYYCFQGSGYPFTFGGGTKVEIKRTVAAPSVFIFPPSDEQLKSGTASVVCLLNNFYPREAKVQW<br>KVDNALQSGNSQESVTEQDSKDSTYSLSSTLTLSKADYEKHKVYACEVTHQGLSSPVTKSFNRGEC
NIST<br>(Heavy chain)	QVTLRESGPALVKPTQTLTLTCTFSGFSLSTAGMSVGWIRQPPGKALEWLADIWWDDKKHYNPSLKDRLTIS<br>KDTSKNQVVLKVTNMDPADTATYYCARDMIFNFYFDVWGQGTTVTVSSASTKGPSVFPLAPSSKSTSGGTA<br>ALGCLVKDYFPEPVTVSWNSGALTSGVHTFPAVLQSSGLYSLSSVVTVPSSSLGTQTYICNVNHKPSNTKVDK<br>RVEPKSCDKTHTCPPCPAPELLGGPSVFLFPPKPKDTLMISRTPEVTCVVVDVSHEDPEVKFNWYVDGVEVH<br>NAKTKPREEQYNSTYRVVSVLTVLHQDWLNGKEYKCKVSNKALPAPIEKTISKAKGQPREPQVYTLPPSREMT<br>KNQVSLTCLVKGFYPSDIAVEWESNGQPENNYKTTPPVLDSDGSFFLYSKLTVDKSRWQQGNVFSCSVMHE<br>ALHNHYTQKSLSLSPGK

Table S2. Summary of unique peptides, PSMs, and MS2 scans observed in each combination of protease and MS fragmentation method. Values represent the median across three replicates.

Protease	MS method	Unique peptides	PSMs	MS2 scans
Trypsin	CID	313	2264	16251
Trypsin	EAD	322	4712	32115
Trypsin	EAciD	339	5728	31765
Chymotrypsin	CID	581	8527	29182
Chymotrypsin	EAD	451	13661	40600
Chymotrypsin	EAciD	420	13429	40497
Vesuvius	CID	1777	6113	32826
Vesuvius	EAD	1541	9919	39401
Vesuvius	EAciD	1675	11005	38497
Krakatoa	CID	1246	3492	25956
Krakatoa	EAD	1943	11472	39725
Krakatoa	EAciD	2118	12236	38359

References

1. Syka, J.E.P., Coon, J.J., Schroeder, M.J., Shabanowitz, J., Hunt, D.F. (2004). Peptide and protein sequence analysis by electron transfer dissociation mass spectrometry. *Proc Natl Acad Sci U S A 26*, 9528–9533. https://doi.org/10.1073/pnas.0402700101.

2. Cooper, H.J. (2005). Investigation of the Presence of b Ions in Electron Capture Dissociation Mass Spectra. *Journal of the American Society for Mass Spectrometry 12*, 1932–1940. https://doi.org/10.1016/j.jasms.2005.07.014.

3. Jones, A.W., and Cooper, H.J. (2010). Probing the mechanisms of electron capture dissociation mass spectrometry with nitrated peptides. *Phys. Chem. Chem. Phys. 41*, 13394–13399. https://doi.org/10.1039/C0CP00623H.

4. Colaert, N., Helsens, K., Martens, L., Vandekerckhove, J., Gevaert, K. (2009). Improved visualization of protein consensus sequences by iceLogo. *Nat Methods 11*, 786–787. https://doi.org/10.1038/nmeth1109-786.

5. McCabe, M.C., Gejji, V., Barnebey, A., Siuzdak, G., Hoang, L.T., et al. (2023). From volcanoes to the bench: Advantages of novel hyperthermoacidic archaeal proteases for proteomics workflows. *J Proteomics*. 104992. https://doi.org/10.1016/j.jprot.2023.104992.

6. Yannone, S.M., Tuteja, V., Goleva, O., Leung, D.Y.M., Stotland, A., et al. (2025). Toward Real-Time Proteomics: Blood to Biomarker Quantitation in under One Hour. *Anal. Chem. 12*, 6418-6426*.* https://doi.org/10.1021/acs.analchem.4c05172.
