# Supplementary material for: Deep coverage and extended sequence reads obtained with a single archaeal protease expedite de novo protein sequencing by mass spectrometry
Source: Cell Syst. 2026 Apr 15;17(4):None. doi: 10.1016/j.cels.2026.101536 (PMC13083267; doi:10.1016/j.cels.2026.101536)
Supplement: Document S2. Transparent peer review records for Pañeda et al [file mmc2.pdf]

Cell Systems
Transparent Peer Review Record

Deep Coverage and Extended Sequence Reads Obtained with a Single Archaeal Protease Expedite *de novo* Protein Sequencing by Mass Spectrometry

Laura Pérez Pañeda, Tereza Kadavá, Tatiana M. Shamorkina, Douwe Schulte, Patrick Pribil, Sibylle Heidelberger, Allison Michele Narlock-Brand, Steven M. Yannone, Joost Snijder, Albert J. R. Heck

Summary

Initial Submission: Received June 30, 2025
Scientific editor: Ernesto Andrianantoandro, Ph.D.
Preprint: https://doi.org/10.1101/2025.05.26.656138

First round of review: Number of reviewers: Three
Three confidential, Zero signed
Revision invited August 11, 2025
Major changes anticipated
Revision received September 17, 2025

Second round of review: Number of reviewers: Three
Three original, Zero new
Three confidential, Zero signed
Accepted January 21, 2026

This Transparent Peer Review Record is not systematically proofread, type-set, or edited. Special characters, formatting, and equations may fail to render properly. Standard procedural text within the

editor's letters has been deleted for the sake of brevity, but all official correspondence specific to the manuscript has been preserved.

Editorial decision letter with reviewers' comments, first round of review

Dear Dr. Heck,

I hope this email finds you well. The reviews of your manuscript are back and I've appended them below. On balance, the reviewers appreciate the goals of the work presented here; they've provided constructive comments that are aligned with our hopes for the paper. Accordingly, we're happy to invite a revision.

I hope you find the reviewer feedback helpful. If you have any questions or concerns, I'm always happy to talk, either over email or by phone. More technical information and advice about resubmission can be found below my signature. Please read it carefully, as it can save substantial time and effort later.

If any changes to the authorship are needed (addition, removal, or a change in order of authors), we require that an authorship change form be provided with the revised manuscript that indicates the reason for the change and provides written consent for the change from all authors, including any that were removed.

I look forward to seeing your revised manuscript.

All the best,

Ernesto Andrianantoandro, Ph.D.
Scientific Editor, Cell Systems

Reviewers' comments:
Reviewer #1: This study investigates the potential of HTA-protease in combination with EAciD fragmentation for MS-based antibody sequencing. The significant coverage achieved within the Complementarity Determining Regions (CDRs) suggests this approach could be a powerful tool for future sequencing of both monoclonal and polyclonal antibodies.

Here are some points I'd like to raise:
1. The Methods section omits the quantities of HTA-proteases used. Without this information, it's impossible to tell if differences in mass spectrometry identification, when compared to trypsin and chymotrypsin, stem from an unfair comparison of enzyme amounts. This crucial detail should be clarified in the publication.

2. When comparing identification numbers across CID, EAD, and EAciD methods, the use of a Byonic score of 150 as a threshold raises concerns. Given that EAciD typically yields a richer array of fragment ions, it may be possible that peptides analyzed with EAciD could more easily surpass this score. Therefore, a direct score comparison might not be entirely equitable. A more robust and impartial scoring criterion, such as the False Discovery Rate (FDR), would be more appropriate.

3. Under a truly fair comparison in antibody sequencing, what is the definitive advantage of HTA-protease over traditional non-specific enzymatic digestion? Quantifying this benefit is essential.

4. In routine antibody analysis, what's the optimal strategy for enzyme use? Should we employ a single HTA-protease, utilize two enzymes in separate MS runs to achieve complementarity, or perhaps combine two enzymes within a single MS run? It's worth exploring whether a mixture of two proteases offers superior results compared to a single one.

5. For de novo antibody sequencing, merely identifying more peptides doesn't necessarily translate to higher accuracy in the final antibody sequences. A proportion of false identifications can misleadingly influence the assembly of peptide sequences. Therefore, it's crucial to demonstrate the real-world performance of this de novo sequencing approach, perhaps by showcasing a detailed example from one of the four mixed antibodies.

Reviewer #2: Manuscript Review

This manuscript details the evaluation of two different thermophilic proteases, named Vesuvius and Krakatoa, and their application for protein sequencing of antibodies. The authors generated general metrics of comparability of these enzymes against the commonly used enzymes trypsin and chymotrypsin across the digestions of 4 different antibody sequences. The enzyme digests were analyzed on newer generation Q-TOF instrument and the authors explored alternative fragmentation modes for increased sequence coverage and identifications of the resulting antibody digests. The authors results were consistent with prior observations that hybrid fragmentation results in the greatest sequence coverage and the peptide sequence matches. The authors present data demonstrating the thermophilic enzymes produce more unique peptides, and longer sequence reads, than trypsin and chymotrypsin. This is somewhat expected given that trypsin and chymotrypsin have more discreet cleavage residues while the thermophilic enzymes have more randomness. This reviewer likes the comparison, but one would wonder if a more applicable comparison would be against a similar random enzyme like pepsin or thermolysin or

at least highlight the high number of sequence reads are a product of the raggedness of peptide cleavage. It is interesting that Vesuvius and Krakatoa are not as similar as one might predict in terms of unique peptides generated. The author argue that a single enzyme could be used protein de novo sequencing. One main point of discussion that does not come across well in the "Single HTA-protease approach ..." section is the author state they are obtaining deep sequence coverage, but there is no real mention, or discussion, of the overall amino acid coverage that was obtained for the various antibodies and how the sequence confirmation is determined at each individual amino acid. The main issue as a lead-in to de novo sequencing is the confirmation of each amino acid in the correct location, preferably with MS/MS fragmentation or increasing peptide length due to enzymatic cleavage. It is not clear how the end fragmentation changes as a result of peptide length and corresponding sequence and if MS/MS, or the enhanced EAD fragmentation on the Q-TOF, provides the necessary sequence information or is just the overlapping peptide sequences, when known, are used for characterization. This discussion would help end reader evaluate the utility of these enzymes for this purpose.

The authors then characterized a mixture of four different antibodies in order to evaluate the applicability toward de novo antibody sequencing. The authors then compared the sequence assemblies of the four antibodies, associated database identifications, sequence alignment scores, and alignment errors with using the thermophilic enzymes. The authors present, for Vesuvius and Krakatoa, the increased sequence reads, general length, and quality MS/MS fragmentation can be applied to de novo sequencing of antibodies. The data presented in Figures 3 and S7 attempt to demonstrate this point. One observation that is not discussed in the main text is the general limited sequence redundancy in the peptide identifications for Cetuximab HC and NIST HC in the CDR3 region using Vesuvius and Krakatoa. This lack in sequence redundancy puts more emphasis on the quality of MS/MS fragmentation for sequence determination and may not be applicable for all antibody sequences. An added discussion of these limited sequence reads is warranted in the main text. It is interesting that the sequence redundancy is antibody sequence dependent, especially as the constant regions of all antibodies are extensively covered. One major issue that is not discussed in the text in any of the characterization sections is the ability to characterize the difference between leucine and isoleucine as they possess the same mass. This is a major issue for de novo sequence analysis as mis-interpretations between those two amino acids can affect the binding affinity when located in an important position. This could be informed through specialized fragmentation on the Q-TOF or enzyme specificity. Distinguishing leucine from isoleucine is easier to resolve using a multiple enzyme strategy due to the variety of obtained peptides. Therefore, demonstrating equivalent performance using a single enzyme would strengthen the argument that a single protease could be the preferred option for this task. The main focus for this issue should center around the variable regions of the light and heavy chains (first ~115 amino acids) or at least a discussion of where the single enzyme approach would have issues. Overall, the data presented is sound and the author's arguments that a single enzyme digest is potentially feasible for de novo antibody sequencing is supported by the included information.

Other suggestions for modifications to the text and figures:
1) Figure S7: Figures would be easier to read with minor tick marks every 50 residues to improve the view of the antibody variable regions.
2) New figure: Adding a supplemental figure(s) showing the combined MS/MS fragmentation for each protein amino acid would make clear the utility of the single enzyme digestion and Q-TOF analysis for

sequencing. It is difficult to discern how much the overlapping peptides or MS/MS is used to obtain the full antibody sequence and overall redundancy of peptide reads.

Reviewer #3: Shamorkina, Paneda, Kadava, et al. present a manuscript describing the use of EAciD tandem MS methods and hyperthermal archaeal proteases for de novo antibody sequencing. The premise and motivation for the study are sound, and overall, it is an interesting and well-executed study that shows the promise of a single-protease approach to antibody sequencing. I have the comments below for the authors to address before this study is suitable for publication.

The authors state "The ultimate goal of this approach is to de novo sequence polyclonal antibody mixtures from bodily fluids, such as blood and saliva," but they use the described workflow to analyze a mixture of four recombinant monoclonal antibodies, stating it is a mimic of an endogenous polyclonal mixture. I do not see four recombinant antibodies being as suitable mimic of a polyclonal antibody mix, so I would ask the authors to support that claim with data or to readjust their claims to saying that this workflow is suitable for simple purified antibody mixtures rather than the complexity of polyclonal antibody pools.

In Figure S2, the authors states that the high number of c and z ions in CID spectra is from secondary fragmentation and neutral losses, but generating 50-75% sequence coverage of peptides across the size range with purely z ions in CID spectra, for example, indicates a high rate of false positives in ion assigns rather than simply neutral losses. This is worrisome for data interpretation overall, especially with the included annotation of w, v, and d ions, and needs to be addressed with statistical rigor before ion assignments for the rest of the data can be believed. How are false ion assignments controlled?

Is it typical for EAD and EAciD methods to generate 50% more MS/MS spectra on the ZenoTOF 7600 system? If so, why? Is it because of the differences in minimum cps thresholds (50 vs 300) used for EAD or CID methods? Adding this to the discussion of why EAD and related methods perform well for de novo antibody sequencing will be helpful.

For Figure 1 annotated spectra, especially sequence coverage with EAciD, what does sequence coverage look like without w, v, and d ions? Including all the ion types makes it somewhat difficult to see what bonds are explained by what ion types.

For Figure S1, how often is 100% sequence coverage achieved vs ions covering the same bonds? Could a category be added to show median overall sequence coverage when combining ion types?

Figure S2 makes it seem that all methods generate >50% sequence coverage with at least 2 ion types across the peptide lengths. How is it that EAciD would be notably superior to the other methods?

Was there a precursor ion charge state dependence observed for peptide fragmentation quality with the methods tested here? The comparisons with longer peptide sequences are interesting and useful, but I am also curious if the longer peptides always carried more charge, or if the EAD and EAciD methods were able to produce useful fragmentation for all charge states.

The authors state "The higher success rate of EAciD identifications reflects the information rich fragmentation spectra", but the success rate (26% vs 28%) and spectra richness as a function of fragment ion types and signal appear to be more or less in line with EAD based on Figure 1A and 1B. If Figure 1C put EAD and EAciD on the same intensity scale (corrected for the one intense ion, like precursor?), would they look that different?

Given the chromatograms in Figure S4 for trypsin and chymotrypsin, it is surprising that chymotrypsin performed so much better than trypsin in terms of PSMs. Do the authors have any thoughts on this? Also, the trypsin, Vesuvius, and Krakatoa chromatograms do not look so different in their high density of well-resolved peaks or peak complexity. Could the authors described how they quantified this in their interpretation that the HTA proteases surpass trypsin?

Do the arrows and asterisks in Figure S4 chromatograms have significant meaning?

Because semi-specific cleavages were used for Krakatoa and Vesuvius proteases, I am curious if semi-specific cleavage settings (or more relaxed settings in general) for trypsin and chymotrypsin would generate more usable identifications that would improve the utility of either canonical protease.

For Figure S9, the data are stated to be from 3 technical replicates. Are data from the combination of all three? If so, what do results from an individual replicate/analysis look like? In general, it would be helpful if the authors could be explicit throughout the manuscript about when data are representing the median or average of replicates to show expected results from a single analysis and when data are cumulative from all three replicates combined.

Authors' response to the reviewers' first round comments

Attached.

Editorial decision letter with reviewers' comments, second round of review

Dear Dr. Heck,

I'm very pleased to let you know that the reviews of your revised manuscript are back, the peer-review process is complete, and only a few minor, editorially-guided changes are needed to move forward towards publication.

Please revise the manuscript to address the final concerns from the reviewers, as well as the suggestions I've made about your manuscript within the "Editorial Notes" section below. Please also include a response to the reviewer comments detailing the changes that were made. Please review these notes along with the detailed formatting requirements listed in the Final Files Checklist. We've also put together this FAQ (click the Final Formatting Checks tab) for your convenience. Please ask any questions you may have, make any necessary changes to your manuscript files, and then upload your final files into Editorial Manager. Once we receive your formatted files, we will go through our formatting checks and let you know if further changes are needed.

We hope to receive your formatted files within 5 business days. Please email me directly if this timing is a problem or you're facing extenuating circumstances. Alternatively, if this manuscript needs to be officially accepted by a particular date because of grant deadlines, applications, or because it will help your trainees, please let me know.

To standardize the referencing style across Cell Press journals, we ask that all in-text citations be formatted as superscripted numbers (e.g. "Multiple reports support this observation.[1,2]"). Moving away from the Harvard referencing style (e.g. Smith *et al*., 2020) will improve author and reader experiences. All manuscripts accepted from now on must use **the superscript numbered Cell Press referencing style**. Make sure to use this numbered referencing style for all new and revised submissions as well. Switching is easy. Just use the updated CSL and EndNote referencing styles for Cell Press articles.

Since there were changes to the authorship (addition, removal, or a change in order of authors), we require that an authorship change form be provided with the revised manuscript that indicates the reason for the change and provides written consent for the change from all authors, including any that were removed.

Below my signature, you'll find specific information about what to expect next regarding formatting checks and working with our Production Department after acceptance. It's been a pleasure working with you, please feel free to contact our journal team with questions.

All the best,

Ernesto Andrianantoandro, Ph.D.
Scientific Editor, Cell Systems

Editorial Notes

Transparent Peer Review:

Thank you for electing to make your manuscript's peer review process transparent. As part of our approach to Transparent Peer Review, we ask that you add the following sentence to the end of your abstract: "A record of this paper's Transparent Peer Review process is included in the Supplemental Information." Note that this ***doesn't*** count towards your 150 word total!

Also, if you've deposited your work on a preprint server, that's great! Please drop me a quick email with your preprint's DOI and I'll make sure it's properly credited within your Transparent Peer Review record.

General:

- Please choose "Methods" as the article type when you upload your revised manuscript
- Please remove the BioRender license document, this is not required. Only a citation of the graphic, per the BioRender user agreement is required:

    All Completed Graphics to be published in any publication (journals, textbooks, websites, etc.) must be accompanied by the following citation either as a caption, footnote or reference for each figure that includes a Completed Graphic:
    " Created in BioRender. Shamorkina, T. (2025) https://BioRender.com/hgpe1k6".

  Since the Graphical Abstract does not have captions, footnotes, or references, please ensure the citation appears in the Graphical Abstract itself (e.g. at the bottom).

Abstract:

Your abstract reads nicely, but is unfortunately too long. Please condense to 150 words or less.

Manuscript Text:

- House style disallows editorializing within the text (e.g. strikingly, surprisingly, importantly, etc.), especially the Results section. These terms are a distraction and they aren't needed—your excellent observations are certainly impactful enough to stand on their own. Please remove these words and others like them. "Notably" is suitably neutral to use once or twice if absolutely necessary.
- We don't allow "priority claims" (e.g. new, novel, etc.). For a discussion of why, read: http://crosstalk.cell.com/blog/getting-priorities-right-with-novelty-claims, http://crosstalk.cell.com/blog/novel-insights-into-priority-claims.

- Please only use the word "significantly" in the statistical sense.

Figures and Legends:

Please look over your figures keeping the following in mind:

- Bar graphs are not acceptable because they obscure important information about the distributions of the underlying data. Please display individual points within your graphs unless their large number obscures the graph's interpretation. In that case, box-and-whisker plots are a good alternative.
- Please ensure that every time you have used a graph, you have defined "n's" specifically and listed statistical tests within your figure legend.
- Please ensure that all figures included in your point-by-point response to the reviewers' comments are present within the final version of the paper, either within the main text or within the Supplemental Information.

Resource Availability:

Please note that Cell Press has recently changed the way it approaches "availability" statements for the sake of ease and clarity. Please revise your resource availability section as follows, noting that the examples used might not pertain to your study. Please note that the Resource Availability section should immediately follow the Discussion section in the main manuscript.

RESOURCE AVAILABILITY

Lead Contact: Further information and requests for resources and reagents should be directed to and will be fulfilled by the Lead Contact, Jane Doe (janedoe@qwerty.com).

Materials Availability: This study did not generate new materials. *-OR-* Plasmids generated in this study have been deposited at [Addgene, name and catalog number]. *-OR-* etc.

Data and Code Availability:

- **Source data statement** (described below)
- **Code statement** (described below)
- Any additional information required to reanalyze the data reported in this paper is available from the lead contact upon request.

Data and Code Availability statements **have three parts and each part must be present. Each part should be listed as a bullet point, as indicated above.**

Instructions for section 1: Data. The statements below may be used in any number or combination, but at least one must be present. They can be edited to suit your circumstance. ***Please ensure that all datatypes reported in your paper are represented in section 1.*** For more information, please consult this list of standardized datatypes and repositories recommended by Cell Press.

• [Standardized datatype] data have been deposited at [datatype-specific repository] and are publicly available as of the date of publication. Accession numbers are listed in the key resources table.

• [Adjective] data have been deposited at [general-purpose repository] and are publicly available as of the date of publication. DOIs are listed in the key resources table.

• [De-identified human/patient standardized datatype] data have been deposited at [datatype-specific repository]. They are publicly available as of the date of publication until [date or delete "until"]. Accession numbers are listed in the key resources table.

• [De-identified human/patient standardized datatype] data have been deposited at [datatype-specific repository], and accession numbers are listed in the key resources table. They are available upon request until [date or delete "until"] if access is granted. To request access, contact [insert name of governing body and instructions for requesting access]. [Insert the following when applicable] In addition, [summary statistics describing these data/processed datasets derived from these data] have been deposited at [datatype-specific repository] and are publicly available as of the date of publication. These accession numbers are also listed in the key resources table.

• Raw [standardized datatype] data derived from human samples have been deposited at [datatype-specific repository], and accession numbers are listed in the key resources table. Local law prohibits depositing raw [standardized datatype] datasets derived from human samples outside of the country of origin. Prior to publication, the authors officially requested that the raw [adjective] datasets reported in this paper be made publicly accessible. To request access, contact [insert name of governing body and instructions for requesting access]. [Insert the following when applicable] In addition, [summary statistics describing these data/processed datasets derived from these data] have been deposited at [datatype-specific repository] and are publicly available as of the date of publication. These accession numbers are also listed in the key resources table.

• The [adjective] data reported in this study cannot be deposited in a public repository because [reason]. To request access, contact [insert name of governing body and instructions for requesting access]. [Insert the following when applicable] In addition, [summary statistics describing these data/processed datasets derived from these data] have been deposited at [datatype-specific or general-purpose repository] and are publicly available as of the date of publication. [Accession numbers or DOIs] are listed in the key resources table.

• This paper analyzes existing, publicly available data. These accession numbers for the datasets are listed in the key resources table.

• [Adjective or all] data reported in this paper will be shared by the lead contact upon request.

Instructions for section 2: Code. The statements below may be used in any number or combination, but at least one must be present. They can be edited to suit your circumstance. ***If you are using GitHub, please follow the instructions here to archive a "version of record" of your GitHub repo at Zenodo, then report the resulting DOI. Additionally, please note that the Cell Systems strongly recommends that you also include an explicit reference to any scripts you may have used throughout your analysis or to generate your figures within section 2.***

• All original code has been deposited at [repository] and is publicly available as of the date of publication. DOIs are listed in the key resources table.

• All original code is available in this paper's supplemental information.

• This paper does not report original code.

Instructions for section 3. Section 3 consists of the following statement: Any additional information required to reanalyze the data reported in this paper is available from the lead contact upon request.

Thank you!

Reviewer comments:

Reviewer #1: This revision has addressed my concerns. The study has demonstrated that combining HTA-proteases with ExD technology will much improve the coverage of peptides, as well as the sequences of CDR3 regions, which is essential to find actionable antibodies towards a specific antigen.

We tested HTA-proteases following the protocol coupled with timsTOF without ExD, the coverage did not much improved comparing to trypsin itself. It is a bit pity that this powerful protease does not work so well

under CID/HCD, this meets the results showed in Fig. S22. Maybe the age of high-speed ExD is comming for Ab sequencing.

Reviewer #2: This manuscript submission is a revised version of the original manuscript. The authors have addressed the major comments from the original review. The resulting manuscript is clear to read and research claims are supported by the associated data.

Minor issues listed below can be addressed for acceptance.
1) Introduction p2. "... two identical heavy (Hc) and two ..." should probably be "... two identical heavy chains (Hc) and two ..." for clarity of defining the Hc acronym.
2) Figure 1. The panel identifiers for B and C are switched and do not reflect the figure legend.
3) Figure 2. The figure legend for panel D is missing an initial explanation sentence similar to what is stated for panel C. Panel D just goes straight into interpretation of the information.
4) Figure S2. Figure legend states y-axis is adjusted to 2e4, but the figure maximum is 6e3.
5) Figure S4. Figure legend still states "... and c- and z-ions in pure CID fragmentation ..." which no longer is depicted in the figure following the manuscript revision.
6) Figure S6. The authors identify w-ions of leucine. Satellite ions are labeled for W4 Leu and W6 Leu, but not labeled for W15 Leu despite showing a flag in the peptide sequence. Also ions for other non-Leu amino acids are depicted with W-ions which could be spurious matches.

Reviewer #3: I thank the reviewers for their thoughtful and thorough responses. They have largely addressed my comments, with only the following requests remaining:

Because two reviewers requested it and it generated interesting information, I request that the authors include Figure R2 in manuscript supplement.

I thank the authors for their thorough investigation into my question about fragment ions and their corrections to their previous method. My question now is about using a static intensity threshold rather than relative percent intensities. A static intensity threshold in arbitrary units can be difficult to translate between experiments, or especially, across different labs. Often a percentage of intensity is more useful. Beyond the 100 au static intensity threshold, could the authors also suggest in the main text what the relative intensity threshold would be that this would translate to for each method? This also would help readers understand the efficiency of dissociation for CID for EAD methods and what S/N may need to be considered for each method.

For their re-analysis of tryptic and chymotryptic data, they show that relaxed search parameters results in less robust data for proteases with known cleavage sites. I ask that they include this point in their manuscript discussion to point out that the semi-specific cleavages used for Krakatoa and Vesuvius also likely inflated some ID numbers at the cost of robustness.

RESPONSE TO REVIEWERS

Reviewers' comments:

Reviewer #1:

This study investigates the potential of HTA-protease in combination with EAciD fragmentation for MS-based antibody sequencing. The significant coverage achieved within the Complementarity Determining Regions (CDRs) suggests this approach could be a powerful tool for future sequencing of both monoclonal and polyclonal antibodies.

Here are some points I'd like to raise:

1. The Methods section omits the quantities of HTA-proteases used. Without this information, it's impossible to tell if differences in mass spectrometry identification, when compared to trypsin and chymotrypsin, stem from an unfair comparison of enzyme amounts. This crucial detail should be clarified in the publication.

We thank the reviewer for assessing our manuscript and pointing out this oversight. The concentration of HTA-proteases used in the study was 8 ng/1 Unit. We revised the manuscript accordingly: “For the HTA digestion, the antibody mixture was diluted into the reducing HTA digestion buffer (1x vendor-provided HTA acidic buffer, 5 mM TCEP, 2 Units (16 ng)/1 µg of protein of Krakatoa protease or 10 Units (80 ng)/1 µg of protein of Vesuvius protease), followed by incubation at 80 °C for 20 min, shaking at 350 rpm.” Of note, the HTA amounts are comparable with trypsin and chymotrypsin used in 20 ng/1 µg protein.

2. When comparing identification numbers across CID, EAD, and EAciD methods, the use of a Byonic score of 150 as a threshold raises concerns. Given that EAciD typically yields a richer array of fragment ions, it may be possible that peptides analyzed with EAciD could more easily surpass this score. Therefore, a direct score comparison might not be entirely equitable. A more robust and impartial scoring criterion, such as the False Discovery Rate (FDR), would be more appropriate

Indeed, the hybrid EAciD fragmentation yields fragment-rich spectra, as we show throughout the manuscript, which is by itself evidently advantageous for *de novo* sequencing. Yet, these denser spectra do not usually result in higher Byonic scores. To support our statement, we included the score distributions of identified PSMs (Fig. R1) showing no major differences in the score distributions between the CID and EAciD PSMs, despite the difference in the fragmentation scheme and resulting fragment ions.

Additionally, we opted for the Byonic scores and |Log Prob| filtering instead of false discovery rate (FDR) calculations because of our experimental design. While FDR calculations are well-established in proteomics of complex samples, they have been shown to yield inaccurate estimates in cases with limited search space, i.e. small number of proteins (N. Gupta, et al., JASMS, 2011). Our mixture of antibodies, with highly overlapping sequences, represents such a case.

Gupta N, Bandeira N, Keich U, Pevzner PA. Target-decoy approach and false discovery rate: when things may go wrong. J Am Soc Mass Spectrom. 2011 Jul;22(7):1111-20. doi: 10.1007/s13361-011-0139-3. Epub 2011 May 5. PMID: 21953092; PMCID: PMC3220955.

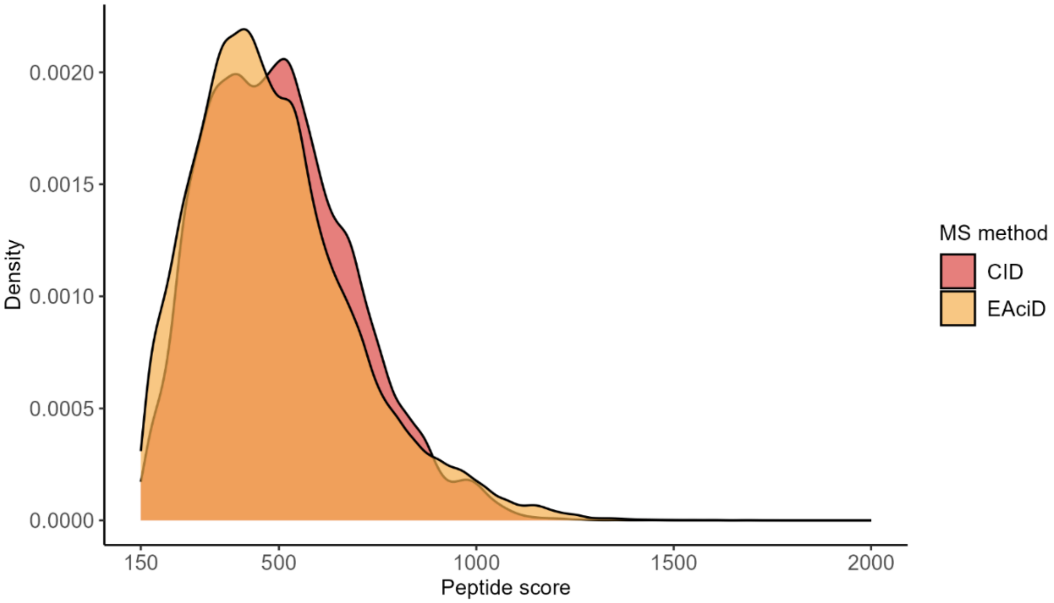

Figure R1 Byonic score distributions of all PSMs for CID and EAciD. Score distribution of CID (red) and EAciD (orange) peptides identified by Byonic with Score > 150 and |Log Prob|> 3, revealing that there is no major difference in the Byonic score distributions.

3. Under a truly fair comparison in antibody sequencing, what is the definitive advantage of HTA-protease over traditional non-specific enzymatic digestion? Quantifying this benefit is essential

To address this key point, we performed new experiments and made a comparison of the HTA-proteases with other less-specific enzymes that are often used for antibody sequencing applications, namely elastase (Gueto-Tettay C., et al., PLOS Computational Biology, 2023) and α-lytic protease (Meyer JG, et al., MCP, 2014). Of note, elastase cleaves C-terminal of A, V, S, G, L, and I, and α-lytic protease cleaves C-terminal of T, A, V, and S. Our new data reveal that neither elastase nor α-lytic can reach the redundancies obtained by the HTA-proteases (Fig. R2). Moreover, even when combining the unique reads obtained by trypsin, chymotrypsin, elastase, and α-lytic protease, each of the individual HTA-proteases outperformed the combination of these four. To us that is a quantifying data result, see Figure R2. This is striking, as each protease from the combination (trypsin, chymotrypsin, elastase, and α-lytic protease) has unique and complementary cleavage preferences. We would like to explore and compare other proteases in the future in another manuscript, and therefore prefer not to use Figure R2 in the current manuscript. Instead, we chose to present just the trypsin and chymotrypsin results in the manuscript, as these proteases are most widely and routinely used in bottom-up workflows, allowing readers to easily assess the potential advantage of HTA-proteases for other applications.

Gueto-Tettay C, Tang D, Happonen L, Heusel M, Khakzad H, et al. (2023) Multienzyme deep learning models improve peptide de novo sequencing by mass spectrometry proteomics. PLOS Computational Biology 19(1): e1010457. https://doi.org/10.1371/journal.pcbi.1010457

Meyer JG, Kim S, Maltby DA, Ghassemian M, Bandeira N, Komives EA. Expanding proteome coverage with orthogonal-specificity α-lytic proteases. Mol Cell Proteomics. 2014 Mar;13(3):823-35. doi: 10.1074/mcp.M113.034710. Epub 2014 Jan 14. PMID: 24425750; PMCID: PMC3945911.

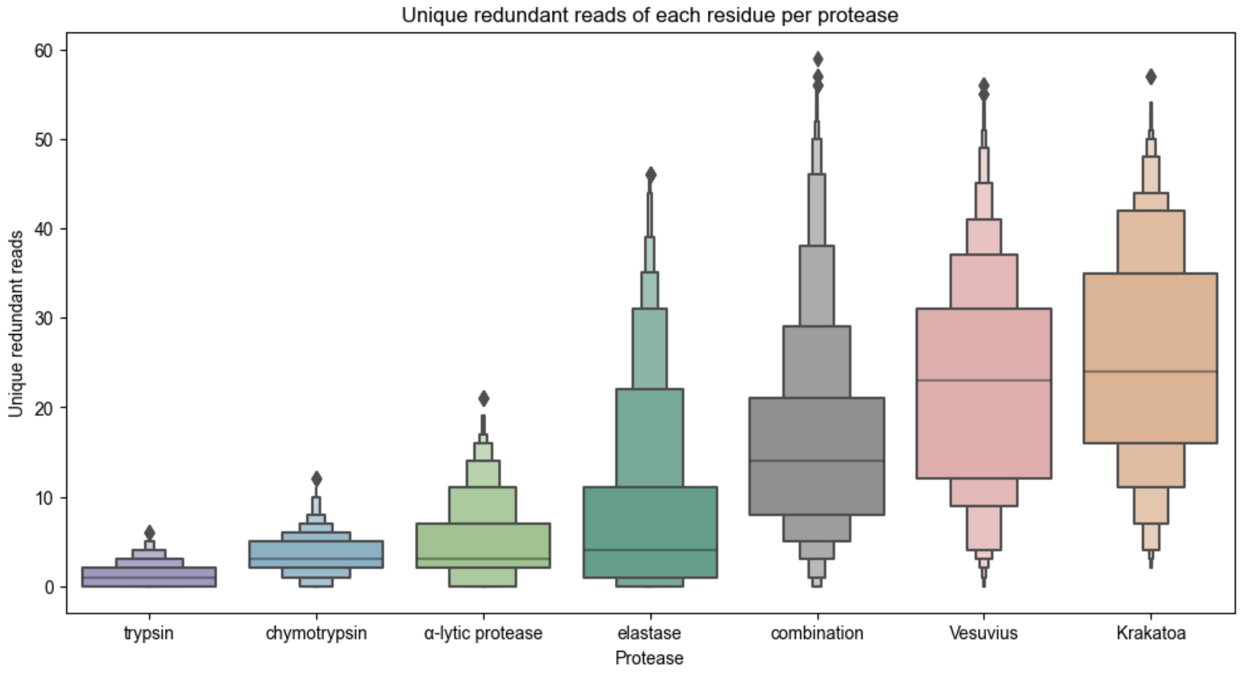

Figure R2. Distribution of the unique redundant sequence reads per protease. Cumulative results from a triplicate analysis, combined visualization for Lc and Hc chains of all four investigated antibodies. The "combination" corresponds to all unique reads detected by trypsin, chymotrypsin, elastase, and α-lytic protease. The median number of redundant sequence reads is 1, 3, 3, 4, 14, 23, and 24 for trypsin (purple), chymotrypsin (blue), α-lytic protease (light green), elastase (dark green), combination (grey), Vesuvius (red), and Krakatoa (orange), respectively. The elastase and α-lytic protease results were analyzed and filtered identically as the HTA-proteases data.

4. In routine antibody analysis, what's the optimal strategy for enzyme use? Should we employ a single HTA-protease, utilize two enzymes in separate MS runs to achieve complementarity, or perhaps combine two enzymes within a single MS run? It's worth exploring whether a mixture of two proteases offers superior results compared to a single one.

We expect that the optimal experimental strategy is dependent on the specific requirements e.g., the amount of material available, the complexity of the sample, cost, and time constraints both for the measurement and sample preparation. Based on our results, performing an independent digestion with both HTA proteases yields a higher number of unique reads per residue in the CDR and thus boosts confidence of the assignment (Figure S16-17). Yet, a single LC-MS run of the Krakatoa digest covered all the CDR regions of mAbs in our sample. Combining the two enzymes might boost the identification and would be worth exploring, but we feel this is beyond the scope of this manuscript. The aim is to show that either Krakatoa or Vesuvius outperform trypsin, chymotrypsin or a combination of these latter two.

5. For de novo antibody sequencing, merely identifying more peptides doesn't necessarily translate to higher accuracy in the final antibody sequences. A proportion of false identifications can misleadingly influence the assembly of peptide sequences. Therefore, it's crucial to demonstrate the real-world performance of this de novo sequencing approach, perhaps by showcasing a detailed example from one of the four mixed antibodies

We indeed agree with the referee that the accuracy of the final antibody sequences is a crucial metric for *de novo* sequencing. Therefore, we determined and included the alignment scores and alignment mistakes, as provided in Fig. 4. This figure highlights that the increased *de novo* redundant reads (Figure 4B) for the HTA-proteases ultimately yield better alignment scores (Figure 4C) and less alignment mistakes (Figure 4D), when compared to trypsin and chymotrypsin. Another metric to assess the *de novo* suitability would be to look at full CDR coverage (new Figure S18) or at the minimum amount of reads per amino acid, which was 0 in case of all proteases, except for Krakatoa, where each amino acid was covered by at least 2 unique reads. The following sentence was added to the manuscript: “Additionally, the HTA-proteases yielded peptides that span both the CDR1-CDR2 regions and cover the full CDR3 region, which aids correct sequence assembly in the de novo analysis (Figure S18)”.

Additionally, we would like to point out that the full Stitch .html reports are included in the PRIDE repository (PXD063988). These reports allow for detailed examination of all the data by everyone:

“How to get to stitch files?

Go to PRIDE and open the html files from Stitch (e.g. “Stitch_KRAK20_EACID_EADsearch.denovo.html”), an interactive report will open, click on Segment Antibody_mix and then on Depth of Coverage Overview, and click on the name of the antibody of interest (e.g. “F59_LC”). Another tab will open with a very detailed view of the alignment mistakes and the peptides used to reconstruct the sequence.”

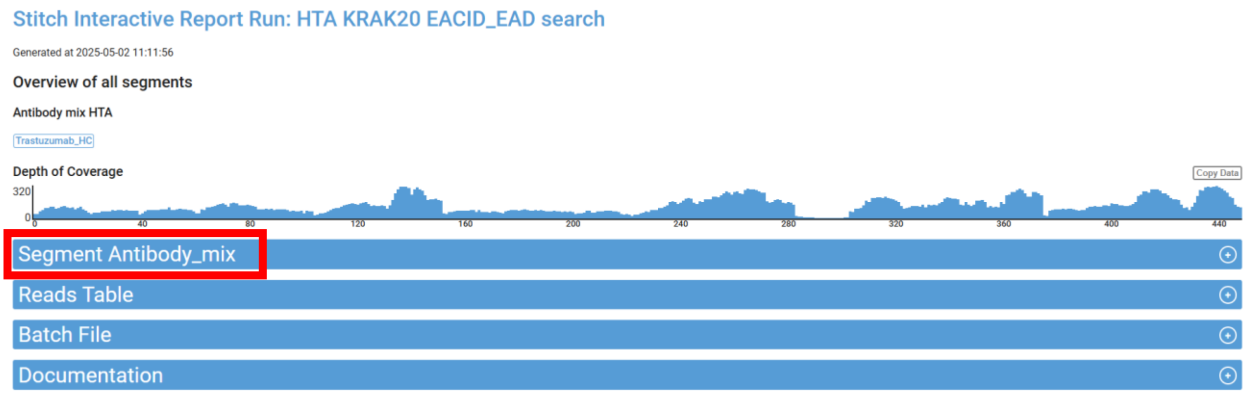

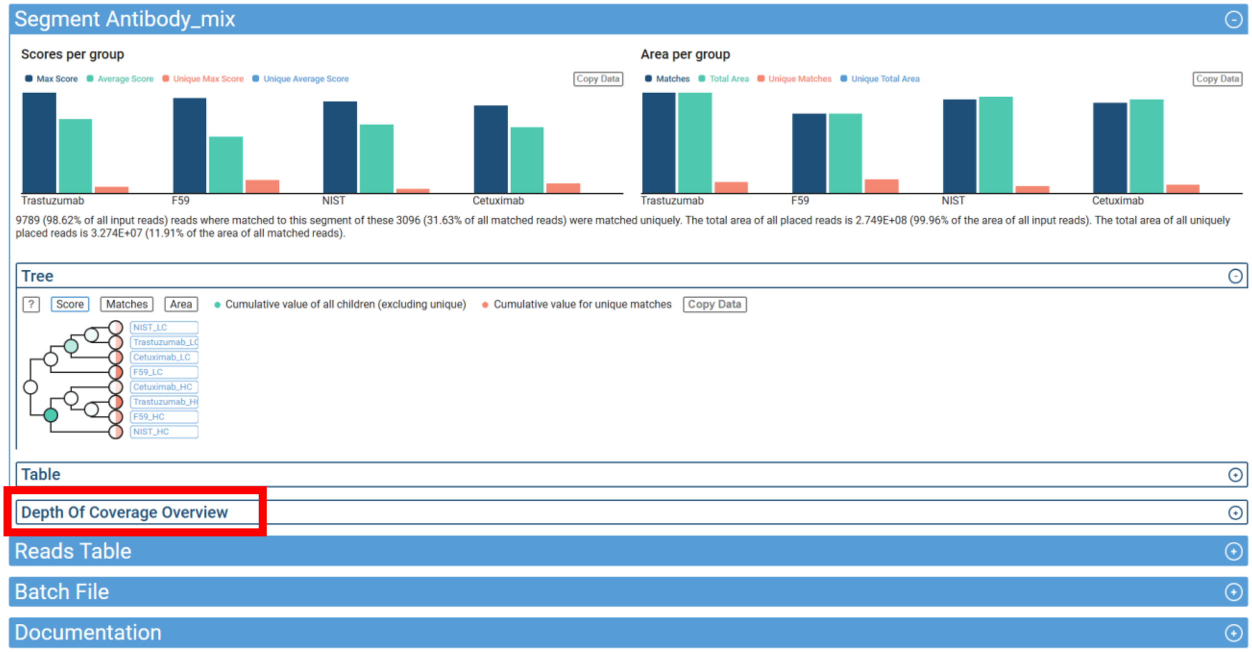

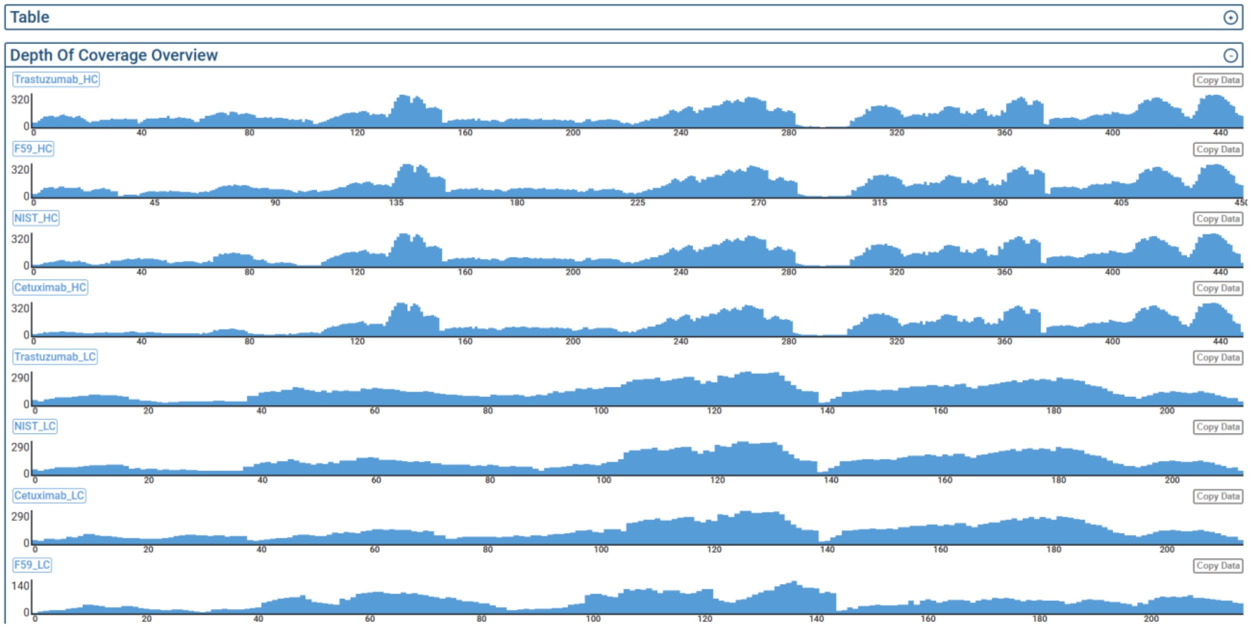

The following sentence was added to the materials and method section to make it clearer how to assess and examine the antibody *de novo* reconstruction data: “The full interactive Stitch .html reports can be examined at the PRIDE repository (PXD063988). In the interactive file, the tab with a detailed view of the alignment mistakes and the peptides used to reconstruct the sequence can be examined by clicking on “Segment Antibody_mix” and then on “Depth of Coverage Overview”, followed by the name of the antibody of interest (e.g. “F59_LC”).”

Reviewer #2: Manuscript Review

This manuscript details the evaluation of two different thermophilic proteases, named Vesuvius and Krakatoa, and their application for protein sequencing of antibodies. The authors generated general metrics of comparability of these enzymes against the commonly used enzymes trypsin and chymotrypsin across the digestions of 4 different antibody sequences. The enzyme digests were analyzed on newer generation Q-TOF instrument and the authors explored alternative fragmentation modes for increased sequence coverage and identifications of the resulting antibody digests. The authors results were consistent with prior observations that hybrid fragmentation results in the greatest sequence coverage and the peptide sequence matches. The authors present data demonstrating the thermophilic enzymes produce more unique peptides, and longer sequence reads, than trypsin and chymotrypsin. This is somewhat expected given that trypsin and chymotrypsin have more discreet cleavage residues while the thermophilic enzymes have more randomness. This reviewer likes the comparison, but one would wonder if a more applicable comparison would be against a similar random enzyme like pepsin or thermolysin or at least highlight the high number of sequence reads are a product of the raggedness of peptide cleavage

We thank the reviewer for evaluating our work. Indeed, we do agree that the benefit of the HTA-proteases is a broad cleavage specificity (or non-specificity) which results in a great number of overlapping peptides. Therefore, we clarified this in the manuscript: “This contrast likely reflects differences in protease cleavage specificities, with trypsin cleaving exclusively C-terminal of K and R, while the HTA-proteases (like chymotrypsin) exhibit broader and less specific cleavage preferences (Figure S7).” Still, there are a few additional advantages of the HTA approach over conventional non-specific digestion, including the low pH, digestion at 80 °C, elimination of an alkylation step (which can introduce sequencing errors during data analysis by generating isobaric species with Gly residues), and the use of LC-MS-compatible buffers, thereby avoiding the need for desalting.

Additionally, as also outlined in the response to reviewer 1, and as suggested by reviewer 2, we compared Krakatoa and Vesuvius with two alike less-specific proteases, namely elastase and α-lytic protease (Figure R2). The comparison shows that elastase and α-lytic protease do not reach the HTA-protease redundancies, neither alone nor when combined with trypsin and/or chymotrypsin, highlighting that the HTA proteases also outperform these less-specific proteases in number of obtained unique peptide-reads.

It is interesting that Vesuvius and Krakatoa are not as similar as one might predict in terms of unique peptides generated. The author argue that a single enzyme could be used protein de novo sequencing. One main point of discussion that does not come across well in the "Single HTA-protease approach ..." section is the author state they are obtaining deep sequence coverage, but there is no real mention, or discussion, of the overall amino acid coverage that was obtained for the various antibodies and how the sequence confirmation is determined at each individual amino acid. The main issue as a lead-in to de novo sequencing is the confirmation of each amino acid in the correct location, preferably with MS/MS fragmentation or increasing peptide length due to enzymatic cleavage.

In this work, we purposely assessed the suitability of the proteases for *de novo* sequencing using redundancy metrics rather than percentage of sequence coverage, as each additional unique read of the same amino acid increases assignment confidence, improves alignment scores, and reduces errors in the final *de novo* sequence. This is reflected in our *de novo* sequencing results (Figure 4C and 4D). Furthermore, the sequence coverage percentages lack information about the confidence of the final sequence, since full coverage can be reached with just a few non-redundant peptides. We agree that this point should be made more explicit in the manuscript to avoid confusion. For completeness, we calculated

the sequence coverage percentages. Both Vesuvius and Krakatoa achieve 100% median sequence coverage, while trypsin and chymotrypsin reach 71% and 93%, respectively and added the information to the manuscript: “In this context, each additional unique read of the same sequence enhances the confidence and increases the accuracy of the assignment of each amino acid. This important feature is not reflected by sequence coverages, which corresponded to a median of 100% for both HTA-proteases, 71% and 93% for trypsin and chymotrypsin, respectively for the four mAb sample.”

Additionally, we present in the new supplementary file a bar chart of alignment mistakes in the variable domains of each antibody (Figure S21), which is a more detailed view of Figure 4C in the manuscript. This highlights the importance of alternative metrics, as high sequence coverage does not necessarily correlate with a lower mistake rate, as illustrated by the trypsin and chymotrypsin data.

Regarding the last part of the question about sequence confirmation and the correctness of each amino acid localization, the antibody sequences were *de novo* reconstructed in Stitch (v1.5), and the consensus sequence for each position is the highest-scoring amino acid for each position. The scores for each amino acid are calculated as the sum of the positional score for all peptides in the alignment, as described in the Stitch manual.

It is not clear how the end fragmentation changes as a result of peptide length and corresponding sequence and if MS/MS, or the enhanced EAD fragmentation on the Q-TOF, provides the necessary sequence information or is just the overlapping peptide sequences, when known, are used for characterization. This discussion would help end reader evaluate the utility of these enzymes for this purpose.
We thank the reviewer for this comment. We highlighted the impact of peptide length on the fragmentation for all three fragmentation techniques in Figure S4, as well as by adding the plot showing peptide coverage by fragment ions for different charge states Figure S5.

Regarding the second part of the question, we think that the advantage of our HTA-EAciD approach lies in the combination of both factors, which allows for sufficient peptide and protein sequence coverage. Firstly, as we show in Figures 1 and S22, EAciD yields better peptide sequence coverage when compared to CID and EAD. Additionally, based on Figures 2-3, and R1, HTA-proteases provide sufficient redundant and overlapping protein sequence coverage, which allows for confident *de novo* assignment without any gaps in the sequence. Thus, the combination of HTA-proteases providing a broad range of highly redundant peptides (high protein sequence coverage) with the EAciD hybrid fragmentation on the ZenoTOF 7600, yielding high peptide sequence coverage, makes this approach stand out.

The authors then characterized a mixture of four different antibodies in order to evaluate the applicability toward de novo antibody sequencing. The authors then compared the sequence assemblies of the four antibodies, associated database identifications, sequence alignment scores, and alignment errors with using the thermophilic enzymes. The authors present, for Vesuvius and Krakatoa, the increased sequence reads, general length, and quality MS/MS fragmentation can be applied to de novo sequencing of antibodies. The data presented in Figures 3 and S7 attempt to demonstrate this point. One observation that is not discussed in the main text is the general limited sequence redundancy in the peptide identifications for Cetuximab HC and NIST HC in the CDR3 region using Vesuvius and Krakatoa. This lack in sequence redundancy puts more emphasis on the quality of MS/MS fragmentation for sequence

determination and may not be applicable for all antibody sequences. An added discussion of these limited sequence reads is warranted in the main text. It is interesting that the sequence redundancy is antibody sequence dependent, especially as the constant regions of all antibodies are extensively covered.

We agree with the reviewer that the CDR3 regions of Cetuximab HC and NIST HC show limited redundancy in peptide identifications. It is important to note that the CDR3 region is usually perceived as the most challenging mAb region to cover because of its high variability, adjacent framework sequence hindering its coverage by trypsin, and its greater length, when compared to the CDR1 and CDR2 regions (Le Bihan, T, et al., 2024, Nat. Commun.; Peng, W, et al., 2021, JPR; Gadush, M V, 2022, JPR). Additionally, analyzing a mixture of four different antibodies further accentuated this challenge, as the signal from all variable CDR regions got diluted by constant region peptides shared among all 4 mAbs. Nevertheless, we were able to recover the CDR3 sequences for all antibodies in the mixture using our HTA approach (Figures 3, 4, S17-S18). Importantly, trypsin and chymotrypsin did not provide the necessary information at all. As we already mentioned above, we think that the better EAciD fragmentation quality (Figures 1, S4-5, S22) contributed to a better overall peptide sequence coverage, aiding confident CDR peptide identifications.

Le Bihan, T., Nunez de Villavicencio Diaz, T., Reitzel, C., Lange, V., Park, M., Beadle, E., Wu, L., Jovic, M., Dubois, R.M., Couzens, A.L., et al. (2024). De novo protein sequencing of antibodies for identification of neutralizing antibodies in human plasma post SARS-CoV-2 vaccination. Nat. Commun. 15, 8790. https://doi.org/10.1038/s41467-024-53105-8.

Peng, W., Pronker, M.F., and Snijder, J. (2021). Mass Spectrometry-Based De Novo Sequencing of Monoclonal Antibodies Using Multiple Proteases and a Dual Fragmentation Scheme. J. Proteome Res. 20, 3559–3566. https://doi.org/10.1021/acs.jproteome.1c00169

Gadush, M.V., Sautto, G.A., Chandrasekaran, H., Bensussan, A., Ross, T.M., Ippolito, G.C., and Person, M.D. (2022). Template-Assisted De Novo Sequencing of SARS-CoV-2 and Influenza Monoclonal Antibodies by Mass Spectrometry. J. Proteome Res. 21, 1616–1627. https://doi.org/10.1021/acs.jproteome.1c00913.

One major issue that is not discussed in the text in any of the characterization sections is the ability to characterize the difference between leucine and isoleucine as they possess the same mass. This is a major issue for de novo sequence analysis as mis-interpretations between those two amino acids can affect the binding affinity when located in an important position. This could be informed through specialized fragmentation on the Q-TOF or enzyme specificity. Distinguishing leucine from isoleucine is easier to resolve using a multiple enzyme strategy due to the variety of obtained peptides. Therefore, demonstrating equivalent performance using a single enzyme would strengthen the argument that a single protease could be the preferred option for this task. The main focus for this issue should center around the variable regions of the light and heavy chains (first ~115 amino acids) or at least a discussion of where the single enzyme approach would have issues. Overall, the data presented is sound and the author's arguments that a single enzyme digest is potentially feasible for de novo antibody sequencing is supported by the included information.

We thank the reviewer for this excellent comment. It has been described before that electron-based fragmentation can generate w- and d-ions that are important for the dissection between isobaric Leu and Ile (Johnson, R S, et al., 1988, Int. J. Mass Spectrom.). This has a clear and important benefit in the antibody

sequencing field (Schulte D, et al., 2024, JPR); however, it was not the major focus of this work. Still, to address this point, we included example spectra of peptides which allow discriminating Leu from Ile as Figure S6 and adjusted the manuscript by adding a sentence: “An additional advantage of EAciD is generation of informative satellite that can be used to distinguish isobaric Leu/Ile (Figure S6) (34).”.

Richard S. Johnson, Stephen A. Martin, Klaus Biemann, Collision-induced fragmentation of (M + H)+ ions of peptides. Side chain specific sequence ions, International Journal of Mass Spectrometry and Ion Processes, 86, 1988, 137-154, ISSN 0168-1176, https://doi.org/10.1016/0168-1176(88)80060-0.

A Handle on Mass Coincidence Errors in De Novo Sequencing of Antibodies by Bottom-up Proteomics, Douwe Schulte and Joost Snijder, Journal of Proteome Research 2024 23 (8), 3552-3559, DOI: 10.1021/acs.jproteome.4c00188

Other suggestions for modifications to the text and figures:

1) Figure S7: Figures would be easier to read with minor tick marks every 50 residues to improve the view of the antibody variable regions.

We added the tick marks in the original Figure S7 (S15 after the revision) as suggested.

2) New figure: Adding a supplemental figure(s) showing the combined MS/MS fragmentation for each protein amino acid would make clear the utility of the single enzyme digestion and Q-TOF analysis for sequencing. It is difficult to discern how much the overlapping peptides or MS/MS is used to obtain the full antibody sequence and overall redundancy of peptide reads.

As we discussed above, we think that the advantage of our HTA-EAciD approach is in the combination of high peptide and protein sequence coverages. The first is facilitated by multiple ion series obtained by EAciD, the latter is achieved through a single HTA-enzyme digestion yielding highly redundant overlapping peptides. To better illustrate these points, we included Fig. S18 and S22 in the revised manuscript.

Reviewer #3:

Shamorkina, Paneda, Kadava, et al. present a manuscript describing the use of EAciD tandem MS methods and hyperthermal archaeal proteases for de novo antibody sequencing. The premise and motivation for the study are sound, and overall, it is an interesting and well-executed study that shows the promise of a single-protease approach to antibody sequencing. I have the comments below for the authors to address before this study is suitable for publication.

We thank the reviewer for assessing our manuscript, and the positive comments on our work.

The authors state "The ultimate goal of this approach is to de novo sequence polyclonal antibody mixtures from bodily fluids, such as blood and saliva," but they use the described workflow to analyze a mixture of four recombinant monoclonal antibodies, stating it is a mimic of an endogenous polyclonal mixture. I do not see four recombinant antibodies being as suitable mimic of a polyclonal antibody mix, so I would ask the authors to support that claim with data or to readjust their claims to saying that this workflow is suitable for simple purified antibody mixtures rather than the complexity of polyclonal antibody pools.

In that statement, we were referring to the ultimate goal of the *de novo* sequencing as a field, rather than to the narrower goal of the manuscript to evaluate new proteases and hybrid fragmentation. We agree that the statement should be adjusted and we revised the manuscript as follows: "The ultimate goal in the *de novo* antibody sequencing field is to retrieve sequences of specific antibodies, from polyclonal antibody mixtures as present in liquid biopsies, such as blood and saliva (15)."

In Figure S2, the authors states that the high number of c and z ions in CID spectra is from secondary fragmentation and neutral losses, but generating 50-75% sequence coverage of peptides across the size range with purely z ions in CID spectra, for example, indicates a high rate of false positives in ion assigns rather than simply neutral losses. This is worrisome for data interpretation overall, especially with the included annotation of w, v, and d ions, and needs to be addressed with statistical rigor before ion assignments for the rest of the data can be believed. How are false ion assignments controlled?

The reviewer raises an important point and got us worried as well: we agree that c/z-ions are very unlikely to be present in CID spectra. We have to admit that this has been an oversight made by us, and thus we appreciate very much that this reviewer 3 brought it up. In our original manuscript we opted for inclusive (too simple) annotation parameters that ended up giving an inflated view of the annotation statistics. This is especially the case for the sequence coverage reported in original Fig S2, as it completely disregards the relative intensity and mass accuracy of the matched fragment ion peaks. As already shown in main Figure 1B, the matched ("miss-annotated") c- and z-fragments in CID spectra are on average more than 10-fold less intense than the corresponding b/y-fragments. We think that the relatively higher intensity for (mis)assigned z-ions, especially, is due to the very common neutral loss of ammonia (-17.026 Da), which is indistinguishable from the relative mass shift between y- and z-ions (-17.025 Da). Conversely, b-ions cannot obtain a neutral gain of +17 Da to match the theoretical c-ion series, so we think the >10-fold lower intensities are an accurate reflection of the level of (noise) peaks that are misassigned.

This original oversight has prompted us to do a thorough reanalysis of all the data and annotations. To address this issue, we tested different raw fragment ion intensity thresholds and ppm mass error filters (Figure R3 and R4), and assessed the fragment ion count decrease under varying threshold conditions. Indeed, we noticed that the number of ions and overall peptide coverage decreased with the increasing intensity threshold. Furthermore, with the increase of the intensity filter, we see a tremendous decrease of annotated c- and z-ions, but only in the CID spectra in comparison to b- and y-ions, which reflects that those earlier assigned c and z ion annotations are likely in the noise. At the intensity threshold higher than 100 (AU), we started to observe also high losses of b- and y-ions together with combined fragment intensity and peptide coverage in CID (Figure R3). In terms of mass tolerance, the highest drop of c- and z-ions in CID is at a threshold of 10 ppm filter (Figure R4). Therefore, we did chose to reevaluate all the data with an intensity threshold of 100 and a stringent 10 ppm mass error and recalculate the ion statistics accordingly. These more stringent filtering conditions considerably reduced the false assignment of c/z ions in CID spectra (loss of 61% of the annotated c- and z- ions, whereas we loosed only 5% of the b- and y-ions), though the issue with ammonia loss of y-ions matching theoretical z-ions remains. Reanalyzing the EAD and EAciD data with the same stringent filters we did not observe substantial losses of annotated b-, c-, y- and z -ions. Additionally, we provide a comparison of the peptide coverage (%) originally provided (Figure R5) and the current data with the 10ppm filter and 100 intensity threshold (Figure R6).

The software used for spectrum annotation has a built-in estimate for false match rate, based on arbitrarily shifting the input spectrum within a 50 Th window and matching the shifted peaks against the same theoretical peptide fragments. It is true that this false match rate increases when a wider scope of fragment types is included. Therefore, additionally to the stringent intensity and ppm error filters, we used the annotator search parameters of CID method containing only b- and y-ions for the reanalysis of the CID data and EAciD method containing b-, c-, y- and z-ions for the EAD and EAciD data. On this note, the eV of 9 represents the hot EAD and therefore, is expected to produce b-and y-ion along with c- and z-ions under EAD parameters. Overall, with the adjusted ions statistics, the message of the manuscript that EAciD method surpasses CID and EAD stands. We adjusted the method section as follows: “For annotation of the fragment ions, thresholds were optimized to reduce noise annotations. The criteria chosen filtered out the majority of “miss-annotated” spurious c- and z- ions in the CID data, while retaining the majority of b- and y- ions. This led to an intensity threshold of 100 and a 10 ppm mass error filter. These settings were used across all datasets generated (CID, EAD, and EAciD) when peaks were assigned by using Annotator (46).   ”.

In line with the results of the filtering evaluation, we reanalyzed all the data with an intensity threshold of 100 and 10 ppm mass error and adjusted Figure 1B, 1C, S1, S2 and S5. As the c- and z- ions in the CID data predominantly fell below our cut-offs in the new analysis we searched the CID data with strict CID parameters, including exclusively b- and y- ions and we did not include c- and z- ions for the reanalysis of the data.

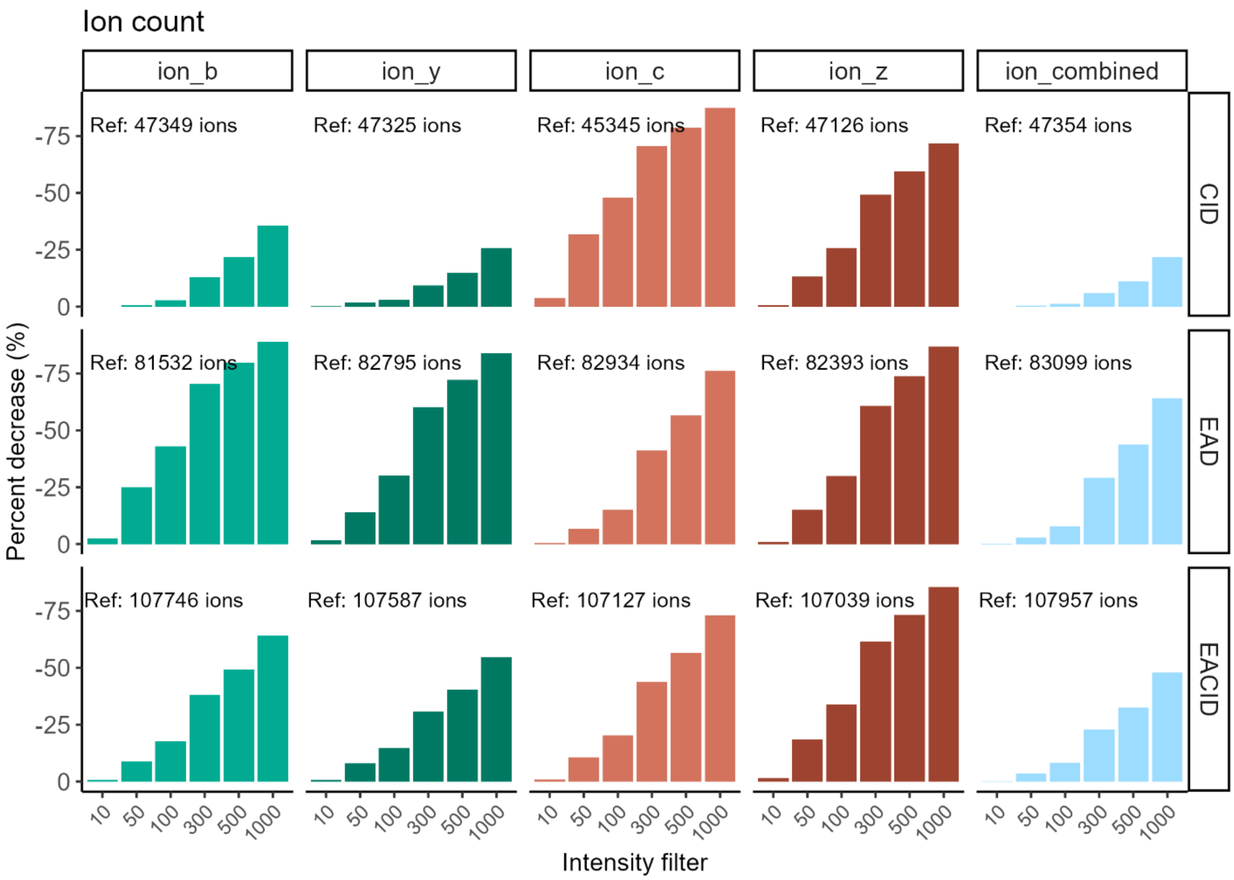

Figure R3. The percentage decrease of the number of ions detected in MS2 spectra depending on the absolute MS2 intensity threshold. The 0 intensity value and 20ppm error was taken as reference for all comparisons. The percentage decrease of the ion count on the y-axes was calculated against the reference values (depicted as Ref on the plots) for all corresponding fragment ions (b-, c-, y-, z- and all ions combined) in CID, EAD and EAciD methods. The Intensity filter on x-axes represents the absolute fragment intensity threshold.

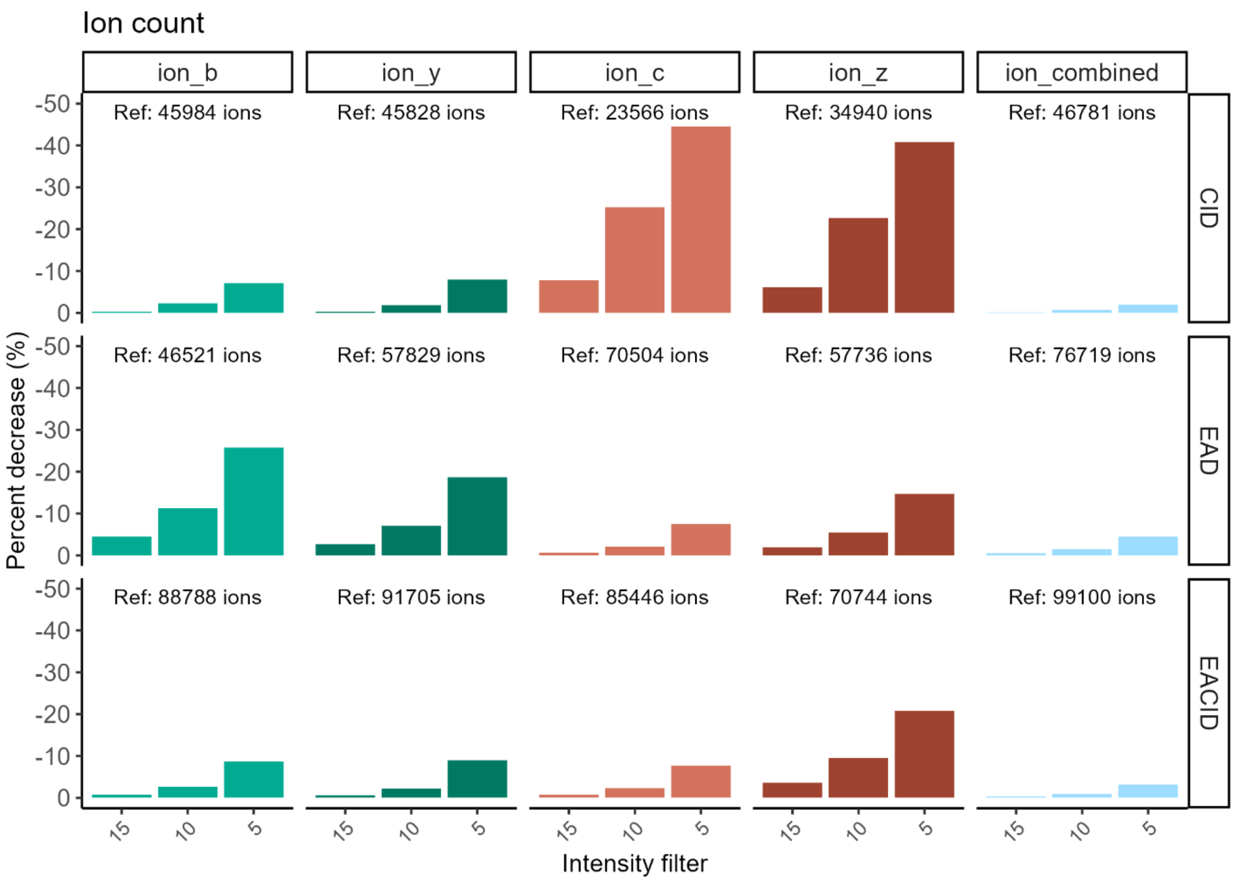

Figure R4. Percentage decrease of the number of ions detected in MS2 spectra depending on the MS2 fragment match tolerance. The 20ppm tolerance and 100 intensity threshold was taken as reference for all comparisons. The percentage decrease of the ion count on the y-axes was calculated against the reference values (depicted as Ref on the plots) for all corresponding fragment ions (b-, c-, y-, z- and all ions combined) in CID, EAD and EAciD methods. The Intensity filter on x-axes represents the absolute fragment intensity threshold.

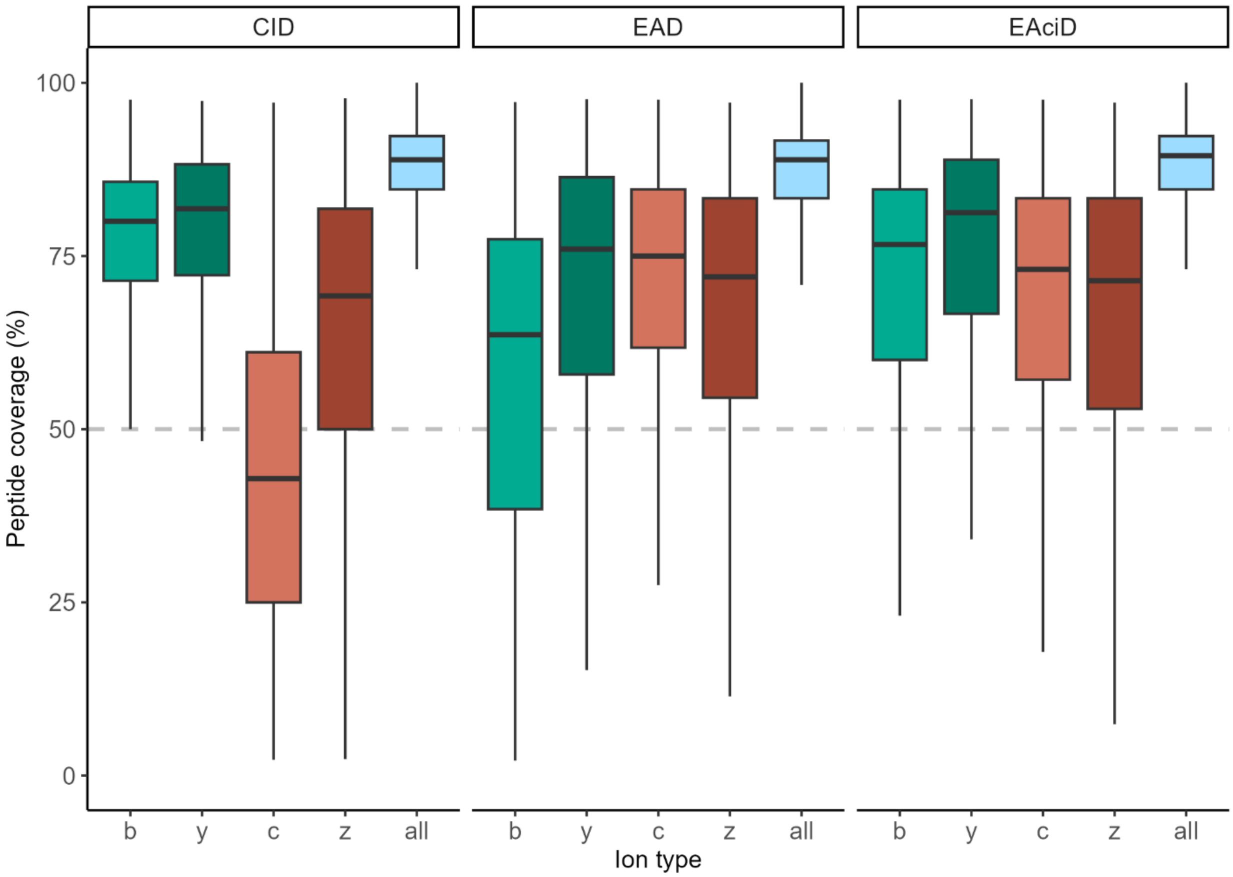

Figure R5. Peptide coverage by different fragment ions obtained by different fragmentation methods, CID, EAD and hybrid EAciD. The peptide coverage percentage represents the initial data calculated with no fragment ion intensity filter and 20ppm mass error tolerance.

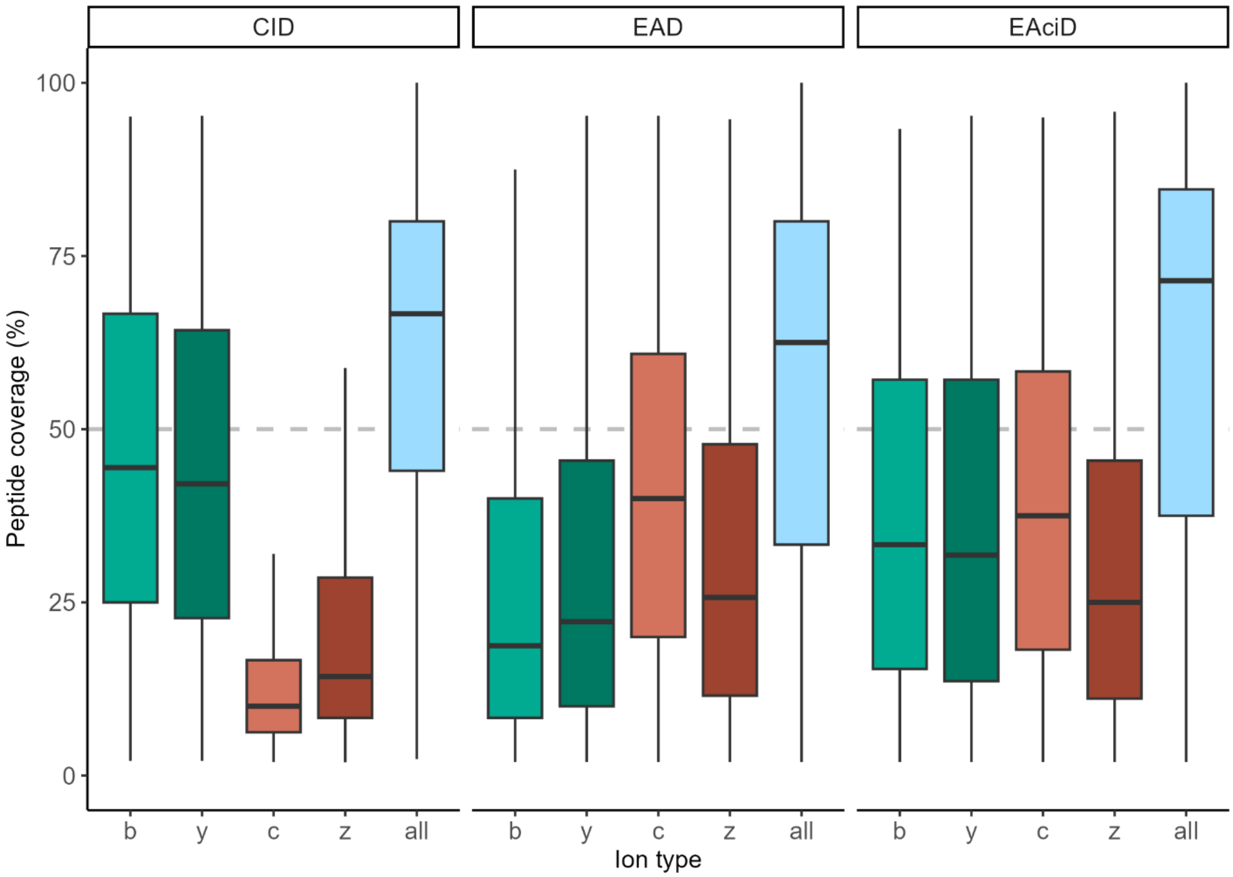

Figure R6. Peptide coverage by different fragment ions obtained by different fragmentation methods, CID, EAD and hybrid EAciD. The peptide coverage percentage represents the data calculated with fragment ion intensity filter of 100 and 10ppm mass error tolerance. The decrease in c- and z-ions and as a result the decrease in peptide coverage by c- and z-ion is clearly observed in CID-generated data after the strict filtering of noise and mass accuracy.

Is it typical for EAD and EAciD methods to generate 50% more MS/MS spectra on the ZenoTOF 7600 system? If so, why? Is it because of the differences in minimum cps thresholds (50 vs 300) used for EAD or CID methods? Adding this to the discussion of why EAD and related methods perform well for de novo antibody sequencing will be helpful.

We agree with the reviewer, the cps counts indeed do influence the number of MS2 events, yet it does not influence observed differences between fragmentation methods. CID fragmentation depends on the charge of the precursor and thus favors highly charged peptides containing lysine and/or arginine residues. This makes CID an excellent choice for tryptic digests, which mainly produce b- and y-ion series. In contrast, EAD methods are less dependent on precursor composition and can generate a broader range of ion series. As a result, they deliver high-quality data even for non-tryptic peptides. This trend is illustrated in a new supplementary Figure S22, where we show that EAciD boosts *de novo* identified unique peptides, especially for non-tryptic digests. Additionally, the slightly longer reaction times and consequently different MS cycle length of the EAD/EAciD methods, the use of lower cps in EAD-based methods is well justified. To clarify this, we expanded the Results and Discussion to address this point, as

the reviewer requested: “Of note, the difference in the amount of MS2 scans is reflective of precursor counts per second (cps) thresholds which varied for CID and EAD-based methods, due to different lengths of the fragmentation events”.

For Figure 1 annotated spectra, especially sequence coverage with EAciD, what does sequence coverage look like without w, v, and d ions? Including all the ion types makes it somewhat difficult to see what bonds are explained by what ion types.

To provide a clearer comparison of the spectra, we included two additional figures in the supplementary information. These show the spectra present in Figure 1C, annotating only a-, b-, c-, x-, y-, and z- ions and adjusting the x axes to 6e3 (new Figure S2) and 1e3 (new Figure S3) intensities, respectively.

For Figure S1, how often is 100% sequence coverage achieved vs ions covering the same bonds? Could a category be added to show median overall sequence coverage when combining ion types?

To answer this question, we added the median overall sequence coverage in Figure S1. The results show that the additional fragments generated by increasing EAD KE yield similar overall peptide coverages. This suggests that the added value of EAciD is mostly in the increased number of fragment ions covering the same bonds. Additionally, 100% peptide sequence coverage by all possible fragments has been achieved for 36, 41, 43, 35, 43, 48, 52, and 77 PSMs for KE in the range of 5-12 eV, respectively.

Figure S2 makes it seem that all methods generate >50% sequence coverage with at least 2 ion types across the peptide lengths. How is it that EAciD would be notably superior to the other methods?

Generally, we observed that CID fragmentation generates b- and y-ions, EAD produces mostly c- and z-ions, and the EAciD method generates a combination of all these fragment types (Fig. 1) (Frese, C K, et al., 2012, Anal. Chem.). Even though all methods reach similar coverages per ion series, the presence of all these series in EAciD allows for complementary confirmations for the same amino acid position within a single spectrum, which is what makes EAciD superior for the *de novo* applications. This is reflected in the new supplementary Figure S22, as mentioned above, where it becomes clear that EAciD provides a broader array of *de novo* peptides than CID, making this even more evident for non-tryptic digests.

Christian K. Frese, A. F. Maarten Altelaar, Henk van den Toorn, Dirk Nolting, Jens Griep-Raming, Albert J. R. Heck, and Shabaz Mohammed, *Analytical Chemistry* **2012** *84* (22), 9668-9673, DOI: 10.1021/ac3025366

Was there a precursor ion charge state dependence observed for peptide fragmentation quality with the methods tested here? The comparisons with longer peptide sequences are interesting and useful, but I am also curious if the longer peptides always carried more charge, or if the EAD and EAciD methods were able to produce useful fragmentation for all charge states.

In terms of the relation between precursor ion charge state and spectral quality, we represented the peptide coverage (%) distribution per charge state for every fragmentation method and ion type. As

illustrated in the new supplementary Figure S5, b- and y-ions show a lower dependency on the precursor ion charge state than c- and z-ions (Macauslane, K L, et al., 2024, Anal. Chem.); the latter ones seem to fragment better at higher charge states.

To address the second part, we plotted the charge state and peptide length distributions per protease and fragmentation technique (new Figure S14). It appears that some longer peptides tend to carry higher charges, but this doesn't seem to be a direct relation. Additionally, we referenced the figure in the manuscript: "These HTA peptides also cover a substantially wider range in both peptide lengths and charges (Figure 2B, S12-S14).".

Electron-Activated Dissociation and Collision-Induced Dissociation Glycopeptide Fragmentation for Improved Glycoproteomics Kyle L. Macauslane, Cassandra L. Pegg, Amanda S. Nouwens, Edward D. Kerr, Joy Seitanidou, and Benjamin L. Schulz Analytical Chemistry 2024 96 (27), 10986-10994, 10.1021/acs.analchem.4c01450

The authors state "The higher success rate of EAciD identifications reflects the information rich fragmentation spectra", but the success rate (26% vs 28%) and spectra richness as a function of fragment ion types and signal appear to be more or less in line with EAD based on Figure 1A and 1B. If Figure 1C put EAD and EAciD on the same intensity scale (corrected for the one intense ion, like precursor?), would they look that different?

We thank the reviewer for the comment. While the difference in MS2 to PSM success rate is rather minor, as the reviewer pointed out, there is a greater difference in the higher confidence PSMs passing the score cutoffs, which results in a final 18% and 24% MS2 to passing PSMs success rate for EAD and EAciD, respectively. This difference is indeed reflective of information-rich spectra, containing multiple fragment series. To make this clear in the manuscript, we adjusted the main text accordingly: "The hybrid EAciD method surpassed both CID and EAD methods in the number of identified and passing PSMs, resulting in 36% MS2 to passing PSMs success rate, compared to 25% and 27% for CID and EAD, respectively.".

Furthermore, to better compare the EAciD with EAD (and CID), we expanded Fig. 1C by the percentage of intensity explained by fragment types and included the total percentage intensity explained for each spectrum, resulting in 11% for CID, 14% for EAD, and 17% for EAciD. Additionally, we added two supplementary figures, with further annotations of Fig. 1C and adjusted the y-axes to 6e3 and 1e3 intensities, respectively (Fig. S2-S3).

Given the chromatograms in Figure S4 for trypsin and chymotrypsin, it is surprising that chymotrypsin performed so much better than trypsin in terms of PSMs. Do the authors have any thoughts on this? Also, the trypsin, Vesuvius, and Krakatoa chromatograms do not look so different in their high density of well-resolved peaks or peak complexity. Could the authors described how they quantified this in their interpretation that the HTA proteases surpass trypsin?

To better illustrate the chromatographic differences across the datasets, we have replaced Figure S4 with total ion chromatograms (TIC) and extracted ion chromatograms (XICs) of all Byonic-identified peptides as retrieved from Skyline after peak integration (Figures S8-11).

While tryptic, Vesuvius, and Krakatoa peptides elute as sharp, well-defined peaks, the chymotryptic peptides in some cases yield very broad peaks. Given that our MS acquisition employs a 6-second dynamic exclusion, precursors that elute over broad peaks will likely be repeatedly selected for fragmentation. This explains why chymotrypsin digests yield a higher number of total PSMs but fewer unique peptides. Regarding the comparison of trypsin and HTA-digests, HTA-digests contain the XICs if identified peptides show a higher density of narrow peaks, when compared to trypsin and chymotrypsin (Figures S8-11).

Do the arrows and asterisks in Figure S4 chromatograms have significant meaning?

For clarity, we have replaced Figure S4 with Figures S8-11 showing TIC and XICs of Byonic-identified peptides for each sample, omitting the SciexOS software-specific elements.

Because semi-specific cleavages were used for Krakatoa and Vesuvius proteases, I am curious if semi-specific cleavage settings (or more relaxed settings in general) for trypsin and chymotrypsin would generate more usable identifications that would improve the utility of either canonical protease.

We thank the reviewer for raising this interesting point. To address this, we performed additional Byonic searches using unspecific cleavage settings for trypsin and chymotrypsin and filtered the data for Byonic score > 150 and logP > 3 (the cut-offs used for all analyses in the manuscript). In terms of identifications, the median number of unique peptides detected across 3 replicates increased in the unspecific search, resulting in 558 and 845 for trypsin and chymotrypsin, respectively. We inspected the peptides, focusing on the cleavage site specificities (Figure R7). We observed that for trypsin, 17 % of the detected cleavage sites were non-tryptic (not lysine, K, or arginine, R), suggesting that the increase in ID numbers comes at the cost of results robustness. This is particularly critical for *de novo* sequencing, where the correct amino acid assignment heavily relies on correct identifications. Increasing the search space will additionally lead to an increase in missed cleavages, which has been previously shown to be one of the main determinants in the accuracy of de novo assignments (McDonnell, K, et al., 2022, CSBJ). Therefore, we applied the conventional search parameters using the known cleavage sites for the enzymes used in this study.

McDonnell, K., Howley, E., and Abram, F. (2022). The impact of noise and missing fragmentation cleavages on *de novo* peptide identification algorithms. Computational and Structural Biotechnology Journal *20*, 1402–1412. https://doi.org/10.1016/j.csbj.2022.03.008.

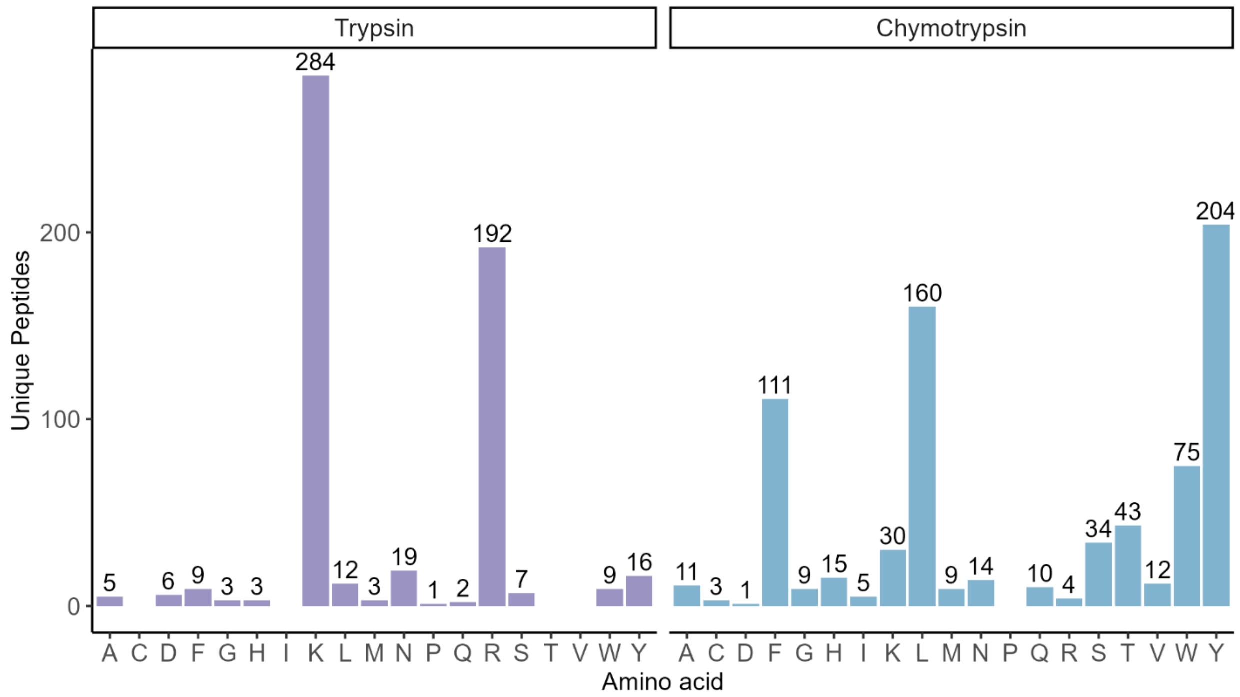

Figure R7. Unique peptides detected in the Byonic search with unspecific cleavage settings. The unspecific search generates additional cleavage sites for trypsin and chymotrypsin, which suggest an increase in misidentifications. This data originates solely from the data generated in EAciD mode, filtered for Byonic score ≥150 and log ≥ 3 from n= 3 technical replicates.

For Figure S9, the data are stated to be from 3 technical replicates. Are data from the combination of all three? If so, what do results from an individual replicate/analysis look like? In general, it would be helpful if the authors could be explicit throughout the manuscript about when data are representing the median or average of replicates to show expected results from a single analysis and when data are cumulative from all three replicates combined.

We thank the reviewer for pointing out this inaccuracy. Indeed, Figure S19 (originally S9) shows the combination of all the unique peptides detected across the 3 replicates. To address the question about the difference between the replicate combination and the individual replicates, we expanded the supplementary information by adding Figure S16, alike Figure 3 from the main text, with just a single replicate. The differences between the two figures are very subtle, showing great redundancies even by a single replicate. Additionally, the difference in using single and multiple replicates is highlighted in Figure S17 (originally S8), showing a rather small added benefit of the replicates. Additionally, we clarified the replicates used for each analysis in the figure captions:

Figure 1. Evaluation of the fragmentation methods, CID, EAD and hybrid EAciD. Data shown in (A) and (B) are cumulative acquired in triplicate for all four used proteases on the four mAb sample. (A) MS2 scans and PSMs for each MS method. The percentages indicate the success rate of the identified MS2 scans (PSMs, light green), as well as the PSMs that meet the cutoff scores selected to filter the Byonic data (PSMs, dark green); Byonic score ≥ 150 and LogProb ≥ 3. EAciD has a higher success rate at both the MS2 scan and the PSM level, outperforming the single fragmentation schemes. (B) Intensity (%) of b-, c-, y-,

and z-ions as observed in each of the fragmentation methods used. EAciD covers optimally all different b-, c-, y- and z-ion types. The percentage of intensity covered for each fragmentation method is 11.27% (9.25e4/8.21e5) for CID; 13.95% (3.07e4/2.20e5) for EAD; and 17.05% (2.12e4/1.25e5) for EAciD. (C) Representative fragmentation spectrum of a 42 amino acid long peptide, originating from the TZB heavy chain, obtained through digestion with Krakatoa. The cartoon at the top of (C) shows the amino acid sequence, whereby always multiple fragment ions (e.g. b-, c-, y-, z-) cover each amino acid throughout the entire peptide. Additional visualizations of the spectra are available as in the Supplementary Figures S2-S3.

Figure 2. Peptide data for the mAb mixture following parallel digestion with the 4 proteases: trypsin (purple), chymotrypsin (blue), Vesuvius (pink), and Krakatoa (orange). (A) Obtained median peptide spectrum matches (PSMs) (left) and median unique peptides (right) identified per protease, median of n=3 replicates. The percentages represent increases in unique peptides, when compared to trypsin. (B) All unique peptides were analyzed based on their charge and peptide length, cumulative results for n=3 replicates. Digestion with Vesuvius and Krakatoa provides substantially broader distributions of peptides both in the peptide length and charge dimension. The ellipses represent the 0.95 confidence level of a multivariate t-distribution. (C) Venn diagram representing the overlap and unique peptides identified by using Vesuvius and Krakatoa, sum of n=3 replicates. (D) The overlapping peptides generated by both Vesuvius and Krakatoa are generally the more abundant ones. This data originates solely from the data generated in EAciD mode, filtered for Byonic score ≥150 and log ≥ 3.
